# Supplementary material for: Quality of evidence in the oral health international data: Contributions for a global profile
Source: PLoS One. 2025 Feb 12;20(2):e0315131. doi: 10.1371/journal.pone.0315131 (PMC11819551; doi:10.1371/journal.pone.0315131)
Supplement: S1 File — (DOCX) [file pone.0315131.s001.docx]

**Supplementary Material**

| Title | Year | Country/WHO area | References |
| --- | --- | --- | --- |
| Early childhood caries and associated risk factors among preschool children in Ras Al-Khaimah, United Arab Emirates | 2017 | United Arab Emirates/ EMRO | Kowash MB, Alkhabuli JO, Dafaalla SA, Shah A, Khamis AH. Early childhood caries and associated risk factors among preschool children in Ras Al-Khaimah, United Arab Emirates. Eur Arch Paediatr Dent. 2017 Apr;18(2):97-103. doi: 10.1007/s40368-017-0278-8. Epub 2017 Feb 27. PMID: 28243836. |
| National Survey of the Oral Health Status of School Children in Dubai, UAE | 2017 | United Arab Emirates/ EMRO | Almashhadani, Shiamaa. (2017). National Survey of the Oral Health Status of School Children in Dubai , UAE. 8. 48-58. |
| Dental caries, fluorosis, and oral health behavior of children from Herat, Afghanistan | 2015 | Afghanistan/ EMRO | Schwendicke F, Doost F, Hopfenmüller W, Meyer-Lueckel H, Paris S. Dental caries, fluorosis, and oral health behavior of children from Herat, Afghanistan. Community Dent Oral Epidemiol. 2015 Dec;43(6):521-31. doi: 10.1111/cdoe.12177. Epub 2015 Jun 18. PMID: 26086211. |
| PAHO - Average Number of Decayed. Missing. or Filled Teeth (DMFT) at 12 Years Old | 2013 | Antigua and Barbuda, Anguilla/ OPAS | <http://new.paho.org/hq/>  index.php?option=com_docman&task=  doc_download&gid=2443&Itemid |
| Dental caries experience among Albanian pre-school children: a national survey | 2017 | Albania/ EURO | Hysi D, Caglar E, Droboniku E, Toti C, Kuscu OO. Dental caries experience among Albanian pre-school children: a national survey. Community Dent Health. 2017 Mar;34(1):46-49. doi: 10.1922/CDH_3940Hysi04. PMID: 28561558. |
| Caries experience and treatment needs among Albanian 12-year-olds | 2014 | Albania/ EURO | Hysi D, Droboniku E, Toti C. Caries experience and treatment needs among Albanian 12-year-olds. Community Dent Health. 2014 Sep;31(3):141-4. PMID: 25300147. |
| Oral health conditions in an Albanian adolescent population: an epidemiological study | 2015 | Albania/ EURO | Laganà, G., Abazi, Y., Beshiri Nastasi, E. et al. Oral health conditions in an Albanian adolescent population: an epidemiological study. BMC Oral Health 15, 67 (2015). https://doi.org/10.1186/s12903-015-0050-6 |
| Fluoride varnish application: a new prophylactic method in Albania. Effect on enamel carious lesions in permanent dentition | 2008 | Albania/ EURO | Xhemnica L, Sulo D, Rroço R, Hysi D. Fluoride varnish application: a new prophylactic method in Albania. Effect on enamel carious lesions in permanent dentition. Eur J Paediatr Dent. 2008 Jun;9(2):93-6. PMID: 18605892. |
| Dental Caries Experience and Oral Health Behaviour Among 12-Year-Olds in the City of Tirana, Albania | 2010 | Albania/ EURO | Hysi, D., Droboniku, E., Toti, E., Xhemnica, L., & Petrela, E. (2010). Dental Caries Experience and Oral Health Behaviour Among 12-Year-Olds in the City of Tirana, Albania. oral health and dental management, 2010, 0-0. |
| Dynamics of dental morbidity in Armenian adults: A national examination survey during 2003-2018 | 2021 | Armenia/ EURO | Mikael Ervand, M., Marina Mitush, M., Izabella Frunze, V., & Gayane Ervand, M. (2021). Dynamics of dental morbidity in Armenian adults: A national examination survey during 2003-2018. Journal of Oral Health and Oral Epidemiology, 10(3), 116-121. doi: 10.22122/johoe.v10i3.1200 |
| Distribution of dental caries and its association with variables of social protection in children 12 years of age in the county of Avellaneda, Province of Buenos Aires | 2017 | Argentina/ OPAS | Fort A, Fuks AJ, Napoli Av, Et Al. Association with variables of social protection in children 12 years of age in the county of Avellaneda, Province of Buenos Aires. Salud Colect. 2017 Jan-Mar;13(1):91-104. Spanish. doi: 10.18294/sc.2017.914. PMID: 28562728. |
| Oral health in 6-year-old schoolchildren from Berisso, Argentina: falling far short of WHO goals | 2010 | Argentina/ OPAS | Llompart G, Marin GH, Silberman M, Merlo I, Zurriaga O; GIS (Grupo Interdisciplinario para Salud). Oral health in 6-year-old schoolchildren from Berisso, Argentina: falling far short of WHO goals. Med Oral Patol Oral Cir Bucal. 2010 Jan 1;15(1):e101-5. doi: 10.4317/medoral.15.e101. PMID: 19680177. |
| Pesquisa Estadual sobre Situação Odontológica 2016: Crianças de seis anos na Áustria | 2016 | Austria/ EURO | Bodenwinkler, Andrea; Sax, Gabriele; Kerschbaum, Hans (2017): Länder-Zahnstatuserhebung 2016: Sechsjährige in Österreich. Zahnstatus sechsjähriger Kinder mit und ohne Migrationshintergrund. Gesundheit Österreich, Wien. |
| A avaliação da condição dos dentes decíduos em crianças de 6 anos no município de Podgorica | 2012 | Austria/ EURO | Duričković, Mirjana & Ivanovic, Mirjana & Popović, Zorica. (2019). The assessment of primary teeth condition in 6 year-old children in Podgorica municipality. Serbian Dental Journal. 66. 15-19. 10.2478/sdj-2019-0002. |
| Pesquisa estadual sobre situação odontológica de 2012 para crianças de 12 anos na Áustria | 2012 | Austria/ EURO | https://jasmin.goeg.at/1308/1/L%C3%A4nder-Zahnstatuserhebung%202012_G%C3%96G.pdf |
| Prevalence of caries in 6-year-old Austrian children | 2012 | Austria/ EURO | Städtler P, Bodenwinkler A, Sax G. Prevalence of caries in 6-year-old Austrian children. Oral Health Prev Dent. 2003;1(3):179-83. PMID: 15641495. |
| Relatório do Instituto Federal Austríaco de Saúde (ÖBIG) | 2003 | Austria/ EURO | <https://repository.publisso.de/resource/frl:470>  7406-1/data |
| The dental health of Australia's children by remoteness: Child Dental Health Survey Australia 2009 | 2009 | Australia/ WPRO | <https://cappmediaprodst.blob.core.windows.net/>  media/2123/ha-d-amarasena-h-crocombe-l-2013-the-dental-health-of-australias-children-by-remoteness-child-dental-health-survey-australia-2009-australian-institute-of-health-and-welfa.pdf |
| The National Child Oral Health Study 2012–14 | 2012 | Australia/ WPRO | <https://www.adelaide.edu.au/press/system/files/>  media/documents/2019-05/ncohs-ebook.pdf |
| Dental health of Australia's teenagers and pre-teen children: the Child Dental Health Survey, Australia 2003-04 | 2003 | Australia/ WPRO | <http://arcpoh.adelaide.edu.au/publications/>  report/statistics/htm_09/DSR_52_CDHS_2003-04.html |
| Australia's dental generations: the National Survey of Adult Oral Health 2004-06 | 2006 | Australia/ WPRO | <http://www.adelaide.edu.au/spdent/dsru/>  data_frame.html |
| Dental caries experience in Aboriginal and Torres Strait Islanders in the Northern Peninsula Area, Queensland | 2007 | Australia/ WPRO | Hopcraft M, Chowt W. Dental caries experience in Aboriginal and Torres Strait Islanders in the Northern Peninsula Area, Queensland. Aust Dent J. 2007 Dec;52(4):300-4. doi: 10.1111/j.1834-7819.2007.tb00506.x. PMID: 18265686. |
| Child Dental Health Survey Australia 2007: 30-year trends in child oral health. | 2012 | Australia/ WPRO | Mejia GC, Amarasena N, Ha DH, Roberts-Thomson KF and Ellershaw AC (2012). Child Dental Health Survey Australia 2007: 30-year trends in child oral health. Dental statistics and research series no. 60. Cat. no. DEN 217. Canberra: Australian Institute of Health and Welfare (internet only). |
| An Investigation into the Prevalence of Dental Caries and its Treatment Among the Adult Population With Low Socio-Economic Status in Baku, Azerbaijan | 2011 | Azerbaijan/ EURO | http://oralhealth.ro/volumes/2011/volume-1/Paper225.pdf |
| The prevalence and severity of early childhood caries in preschool children in the Federation of Bosnia and Herzegovina | 2016 | Bosnia and Herzegovina/ EURO | Šačić L, Marković N, Arslanagić Muratbegović A, Zukanović A, Kobašlija S. The prevalence and severity of early childhood caries in preschool children in the Federation of Bosnia and Herzegovina. Acta Med Acad. 2016 May;45(1):19-25. doi: 10.5644/ama2006-124.152. PMID: 27284794. |
| Caries prevalence of children and adolescents in Bosnia and Herzegovina | 2013 | Bosnia and Herzegovina/ EURO | Markovic N, Arslanagic Muratbegovic A, Kobaslija S, Bajric E, Selimovic-Dragas M, Huseinbegovic A. Caries prevalence of children and adolescents in Bosnia and Herzegovina. Acta Med Acad. 2013 Nov;42(2):108-16. doi: 10.5644/ama2006-124.79. PMID: 24308391. |
| Primeiro exame de saúde bucal na Bélgica. | 2012 | Belgium/ EURO | http://www.riziv.be/homefr.htm |
| Population-Based Prevalence of Oral Conditions as a Basis for Planning Community-Based Interventions: An Epidemiological Study From Rural Burkina Faso | 2021 | Burkina Faso/ AFRO | Clauss A, Sie A, Zabre P, Schmoll J, Sauerborn R, Listl S. Population-Based Prevalence of Oral Conditions as a Basis for Planning Community-Based Interventions: An Epidemiological Study From Rural Burkina Faso. Front Public Health. 2021 Jul 1;9:697498. doi: 10.3389/fpubh.2021.697498. PMID: 34277555; PMCID: PMC8280293. |
| Erratum to: Proceedings of a workshop, held in Constanta, Romania on 22 May 2014, on Oral Health of Children in the Central and Eastern European Countries in the context of the current economic crisis | 2016 | Bulgaria/Hungary/Czech Republic/Lithuania/Latvia/ Moldova, Republic of/ EURO | Hysi D, Eaton KA, Tsakos G, Vassallo P, Amariei C; DPH Group. Erratum to: Proceedings of a workshop, held in Constanta, Romania on 22 May 2014, on Oral Health of Children in the Central and Eastern European Countries in the context of the current economic crisis. BMC Oral Health. 2016 Sep 15;16(1):96. doi: 10.1186/s12903-016-0269-x. Erratum for: BMC Oral Health. 2016 Jul 25;16 Suppl 1:69. doi: 10.1186/s12903-016-0223-y. PMID: 27634476; PMCID: PMC5025609. |
| Comparative study of dental caries and dental fluorosis in populations of different dental fluorosis prevalence | 2009 | Bulgaria/ EURO | Kukleva MP, Kondeva VK, Isheva AV, Rimalovska SI. Comparative study of dental caries and dental fluorosis in populations of different dental fluorosis prevalence. Folia Med (Plovdiv). 2009 Jul-Sep;51(3):45-52. PMID: 19957563. |
| Dental status and associated factors in a dentate adult population in bulgaria: a cross-sectional survey. | 2012 | Bulgaria/ EURO | Damyanov ND, Witter DJ, Bronkhorst EM, Creugers NH. Dental status and associated factors in a dentate adult population in bulgaria: a cross-sectional survey. Int J Dent. 2012;2012:578401. doi: 10.1155/2012/578401. Epub 2012 May 13. PMID: 22654908; PMCID: PMC3359670. |
| Dental News: Oral health Survey in Bahrain | 2016 | Bahrain/ EMRO | <https://issuu.com/tonydib/docs/june_2016_>  dental_news_issuuu |
| Health in Review An International Comparative Analysis of Bermuda Health System Indicators | 2012 | Bermuda/ OPAS | <https://www.gov.bm/sites/default/files/Hea>  lthinReviewfullreport110207_0.pdf |
| Dental workforce development as part of the oral health agenda for Brunei Darussalam | 2013 | Brunei Darussalam/ WPRO | Wilson NH, Shamshir ZA, Moris S, Slater M, Kok EC, Dunne SM, Said SH, Lee JM, Gallagher JE. Dental workforce development as part of the oral health agenda for Brunei Darussalam. Int Dent J. 2013 Feb;63(1):49-55. doi: 10.1111/idj.12005. Epub 2013 Jan 24. PMID: 23410022; PMCID: PMC9374916. |
| Levantamento epidemiológico na Bolívia: CPOD e CEOD para 2015 | 2015 | Bolivia/ OPAS | <https://www.minsalud.gob.bo/component/>  jdownloads/?task=download.send&id=274  &catid=23&m=0&Itemid=646 |
| Pesquisa Nacional de Saúde Bucal- SB Brasil 2010 | 2010 | Brazil/ OPAS | <http://189.28.128.100/dab/docs/geral/>  projeto_sb2010_relatorio_final.pdf |
| Pesquisa Estadual de Saúde Bucal-São Paulo | 2015 | Brazil/ OPAS | <https://www.saude.sp.gov.br/resources/>  ses/perfil/profissional-da-saude/areas-tecnicas-da-ses/e_book_relatorio_sb_sp_2015.pdf |
| Trends in dental caries rates in 12- and 13-year-old schoolchildren from Florianópolis (Brazil) between 1971 and 2005 | 2005 | Brazil/ OPAS | Souza ML, Bastos JL, Peres MA. Trends in dental caries rates in 12- and 13-year-old schoolchildren from Florianópolis (Brazil) between 1971 and 2005. Oral Health Prev Dent. 2006;4(3):187-92. PMID: 16961027. |
| Dental caries in 12-year-old schoolchildren and its relationship with socioeconomic and behavioural variables | 2007 | Brazil/ OPAS | Pereira SM, Tagliaferro EP, Ambrosano GM, Cortelazzi KL, Meneghim Mde C, Pereira AC. Dental caries in 12-year-old schoolchildren and its relationship with socioeconomic and behavioural variables. Oral Health Prev Dent. 2007;5(4):299-306. PMID: 18173091. |
| Declining caries rate in a municipality in northwestern São Paulo State, Brazil, 1998-2004 | 2004 | Brazil/ OPAS | Martins RJ, Garbin CA, Garbin AJ, Moimaz SA, Saliba O. Declínio da cárie em um município da região noroeste do Estado de São Paulo, Brasil, no período de 1998 a 2004 [Declining caries rate in a municipality in northwestern São Paulo State, Brazil, 1998-2004]. Cad Saude Publica. 2006 May;22(5):1035-41. Portuguese. doi: 10.1590/s0102-311x2006000500016. Epub 2006 Apr 28. PMID: 16680356. |
| Social determinants of health and dental caries in Brazil: a systematic review of the literature between 1999 and 2010 | 2010 | Brazil/ OPAS | Boing AF, Bastos JL, Peres KG, Antunes JLF, Peres MA. Social determinants of health and dental caries in Brazil: a systematic review of the literature between 1999 and 2010. Rev bras epidemiol [Internet]. 2014;17:102–15. Available from: https://doi.org/10.1590/1809-4503201400060009 |
| Report of the Chief Medical Officer Dr. Merceline Dahl-Regis Commonwealth of The Bahamas | 2004 | Bahamas/ OPAS | <https://www.bahamas.gov.bs/wps/wcm/connect>  /9209c493-a088-4c03-b3f7-d8c344a53d4e/Chief+Medical+Officer%27s+  Report+2004+-+2008.pdf?MOD=AJPERES |
| Oral Health Status of 3- to 5 -Year-old Children Attending Early Childhood Care and Development Centers in Bhutan: A Pilot Study | 2020 | Bhutan/ SEARO | Phurpa, D., Ngedup, . S., Pem, D., & Alice Lee, M. (2021). Oral Health Status of 3- to 5 -Year-old Children Attending Early Childhood Care and Development Centers in Bhutan: A Pilot Study: Oral Health Status of 3- to 5 -Year-old Children Attending Early Childhood Care and Development Centers in Bhutan: A Pilot Study. Bhutan Health Journal, 6(2), 19–26. https://doi.org/10.47811/bhj.105 |
| Oral health and associated factors in 12 year-old children in Thimphu, Bhutan | 2009 | Bhutan/ SEARO | Ngedup S, LeelataweewudP, Lexomboon D. Oral health and associated factors in 12 year-old children in Thimphu, Bhutan. Int Dent J Paed Dent. 2009; 19 (suppl.1):36. |
| Systems for the provision of oral health care in the black sea countries. Part 12: Ukraine | 2012 | Belarus/ EURO | Marino B, Domenico D, Igmazia C, Denga A, Paganelli C, Dianiskova S. Systems for the provision of oral health care in the black sea countries. Part 12: Ukraine. Oral Health Dent Manag. 2012 Dec;11(4):149-51. Erratum in: Oral Health Dent Manag. 2013 Jun;12(2):119. PMID: 23208591. |
| Popularization of a healthy lifestyle and prevention of diseases within the volunteer activity | 2020 | Belarus/ EURO | Rubtsov E.I., Juraeva Sh.F., Petrova V.O., Kholikova A.A., Bobokalonov R.V. Popularization of a healthy lifestyle and prevention of diseases within the volunteer activity. Endodontics Today. 2020;18(1):65-69. (In Russ.) https://doi.org/10.36377/1683-2981-2020-18-1-65-69 |
| A comparative study of the health care systems of Canada and Saudi Arabia: lessons and insights | 2009 | Canada/ OPAS | Qutub AF, Al-Jewair TS, Leake JL. A comparative study of the health care systems of Canada and Saudi Arabia: lessons and insights. Int Dent J. 2009 Oct;59(5):277-83. PMID: 19998662. |
| Report on the findings of the Oral Health Component of the Canadian health measures survey 2007–2009. | 2007 | Canada/ OPAS | H34-221-2010-eng.pdf |
| Caries experience and related factors in 4-6 year-olds attending dental clinics in Kinshasa, DR of Congo | 2013 | Congo Republic/ AFRO | Songo BF, Declerck D, Vinckier F, Mbuyi MD, Pilipili CM, Kayembe KP. Caries experience and related factors in 4-6 year-olds attending dental clinics in Kinshasa, DR of Congo. Community Dent Health. 2013 Dec;30(4):257-62. PMID: 24575530. |
| Changes in dental caries in Zurich school-children over a period of 45 years | 2010 | Switzerland / EURO | Steiner M, Menghini G, Marthaler TM, Imfeld T. Changes in dental caries in Zurich school-children over a period of 45 years. Schweiz Monatsschr Zahnmed. 2010;120(12):1084-1104. English, German. PMID: 21243546. |
| Caries experience in 7-, 12-, and 15-year-old schoolchildren in the canton of Basel-Landschaft, Switzerland, from 1992 to 2011 | 2011 | Switzerland / EURO | Waltimo T, Menghini G, Weber C, Kulik EM, Schild S, Meyer J. Caries experience in 7-, 12-, and 15-year-old schoolchildren in the canton of Basel-Landschaft, Switzerland, from 1992 to 2011. Community Dent Oral Epidemiol. 2016 Jun;44(3):201-8. doi: 10.1111/cdoe.12206. Epub 2015 Dec 28. PMID: 26709202. |
| Caries prevalence in 2-year-old children in the city of Zurich | 2008 | Switzerland / EURO | Menghini G, Steiner M, Thomet E, Roos M, Imfeld T. Caries prevalence in 2-year-old children in the city of Zurich. Community Dent Health. 2008 Sep;25(3):154-60. PMID: 18839721. |
| Cook Islands National Oral Health Survey 2014-2018 | 2014 | Coco Island/ WPRO | https://www.health.gov.ck/wp-content/uploads/2020/06/Cook-Islands-National-Oral-Health-Strategy-2014.pdf |
| Oral health disparities among adolescents from urban and rural communities of central Chile | 2018 | Chile/ OPAS | Giacaman RA, Bustos IP, Bazán P, Mariño RJ. Oral health disparities among adolescents from urban and rural communities of central Chile. Rural Remote Health. 2018 Apr;18(2):4312. doi: 10.22605/RRH4312. Epub 2018 Apr 16. PMID: 29656652. |
| Impact of rurality on the oral health status of 6-year-old children from central Chile: the EpiMaule study | 2015 | Chile/ OPAS | Giacaman RA, Bustos IP, Bravo-León V, Mariño RJ. Impact of rurality on the oral health status of 6-year-old children from central Chile: the EpiMaule study. Rural Remote Health. 2015 Apr-Jun;15(2):3135. Epub 2015 Jun 25. PMID: 26108477. |
| Dental caries prevalence and tooth loss in chilean adult population: first national dental examination survey | 2012 | Chile/ OPAS | Urzua I, Mendoza C, Arteaga O, Rodríguez G, Cabello R, Faleiros S, Carvajal P, Muñoz A, Espinoza I, Aranda W, Gamonal J. Dental caries prevalence and tooth loss in chilean adult population: first national dental examination survey. Int J Dent. 2012;2012:810170. doi: 10.1155/2012/810170. Epub 2012 Dec 18. PMID: 23316234; PMCID: PMC3536045. |
| Plan Nacional de Salud Bucal 2018-2030 | 2018 | Chile/ OPAS | http://www.odontopediatria-v.cl/site/wp-content/uploads/2018/03/PLAN-NACIONAL-DE-SALUD-BUCAL-2018-2030.pdf |
| Oral Health Practices And Status Of 12-Year-Old Pupils In The Western Region Of Cameroon | 2020 | Cameroon/ AFRO | Agbor, A. M., & Kuimo, T. R. (2020). Oral Health Practices And Status Of 12-Year-Old Pupils In The Western Region Of Cameroon. European Journal of Dental and Oral Health, 1(1). https://doi.org/10.24018/ejdent.2020.1.1.1 |
| Analysis of caries status among 5-year-old children in Shanghai from 2008 to 2011 | 2011 | China/ WPRO | Li CR, Zeng XL, Wang X, Xu W, Chen X. [Analysis of caries status among 5-year-old children in Shanghai from 2008 to 2011]. Shanghai Kou Qiang Yi Xue. 2012 Aug;21(4):451-4. Chinese. PMID: 23135124. |
| Permanent Teeth Caries Status of 12- to 15-year-olds in China: Findings from the 4th National Oral Health Survey | 2018 | China/ WPRO | Quan JK, Wang XZ, Sun XY, Yuan C, Liu XN, Wang X, Feng XP, Tai BJ, Hu Y, Lin HC, Wang B, Si Y, Wang CX, Wang WJ, Rong WS, Zheng SG. Permanent Teeth Caries Status of 12- to 15-year-olds in China: Findings from the 4th National Oral Health Survey. Chin J Dent Res. 2018;21(3):181-193. doi: 10.3290/j.cjdr.a41080. PMID: 30255169. |
| Oral health status in Sichuan Province: findings from the oral health survey of Sichuan, 2015-2016 | 2017 | China/ WPRO | Yin W, Yang YM, Chen H, Li X, Wang Z, Cheng L, Yin QD, Fang HZ, Fei W, Mi FL, Nie MH, Hu T, Zhou XD. Oral health status in Sichuan Province: findings from the oral health survey of Sichuan, 2015-2016. Int J Oral Sci. 2017 Mar;9(1):10-15. doi: 10.1038/ijos.2017.6. PMID: 28358035; PMCID: PMC5379165. |
| Dental Caries Status and its Associated Factors among 3- to 5-year-old Children in China: A National Survey | 2018 | China/ WPRO | Du MQ, Li Z, Jiang H, Wang X, Feng XP, Hu Y, Lin HC, Wang B, Si Y, Wang CX, Zheng SG, Liu XN, Rong WS, Wang WJ, Tai BJ. Dental Caries Status and its Associated Factors among 3- to 5-year-old Children in China: A National Survey. Chin J Dent Res. 2018;21(3):167-179. doi: 10.3290/j.cjdr.a41076. PMID: 30255168. |
| Dental Caries in Chinese Elderly People: Findings from the 4th National Oral Health Survey | 2018 | China/ WPRO | Gao YB, Hu T, Zhou XD, Shao R, Cheng R, Wang GS, Yang YM, Li X, Yuan B, Xu T, Wang X, Feng XP, Tai BJ, Hu Y, Lin HC, Wang B, Si Y, Wang CX, Zheng SG, Liu XN, Rong WS, Wang WJ, Yin W. Dental Caries in Chinese Elderly People: Findings from the 4th National Oral Health Survey. Chin J Dent Res. 2018;21(3):213-220. doi: 10.3290/j.cjdr.a41077. PMID: 30255172. |
| Oral health in China--trends and challenges | 2011 | China/ WPRO | Hu DY, Hong X, Li X. Oral health in China--trends and challenges. Int J Oral Sci. 2011 Jan;3(1):7-12. doi: 10.4248/IJOS11006. PMID: 21449210; PMCID: PMC3469869. |
| Prevalence and Correlates of Dental Caries in an Elderly Population in Northeast China | 2013 | China/ WPRO | Liu L, Zhang Y, Wu W, Cheng M, Li Y, Cheng R (2013) Prevalence and Correlates of Dental Caries in an Elderly Population in Northeast China. PLoS ONE 8(11): e78723. https://doi.org/10.1371/journal.pone.0078723 |
| Analysis of caries status among 5-year-old children in Shanghai from 2008 to 2011 | 2011 | China/ WPRO | Li CR, Zeng XL, Wang X, Xu W, Chen X. [Analysis of caries status among 5-year-old children in Shanghai from 2008 to 2011]. Shanghai Kou Qiang Yi Xue. 2012 Aug;21(4):451-4. Chinese. PMID: 23135124. |
| Dental caries and treatment needs in 12-year-old schoolchildren from public schools of the municipality of Rionegro (Antioquia, Colombia), 2010 | 2010 | Colombia/ OPAS | Vivares-Builes, A. M., Saldarriaga-Saldarriaga, A. F., Muñoz-Pino, N., Miranda-Galvis, M., Colorado-Colorado, K., Montoya-Zuluaga, Y. P. ., Viñas-Sarmiento, Y., & Agudelo-Suárez, A. A. (2012). Dental caries and treatment needs in 12-year-old schoolchildren from public schools of the municipality of Rionegro (Antioquia, Colombia), 2010. Revista Facultad De Odontología Universidad De Antioquia, 23(2), 292–305. https://doi.org/10.17533/udea.rfo.10448 |
| IV Estudio Nacional de Salud Bucal | 2014 | Colombia/ OPAS | <https://www.minsalud.gov.co/sites/>  rid/Lists/BibliotecaDigital/RIDE/VS/PP/  ENT/abc-salud-bucal.pdf |
| Systems for the provision of oral health care in the black sea countries part 13: cyprus | 2013 | Cyprus/ EURO | Charalambous C, Theodorou M. Systems for the provision of oral health care in the black sea countries part 13: cyprus. Oral Health Dent Manag. 2013 Mar;12(1):3-8. PMID: 23474575. |
| Caries prevalence in 12-year-old Cypriot children | 2012 | Cyprus/ EURO | Panagidis D, Schulte AG. Caries prevalence in 12-year-old Cypriot children. Community Dent Health. 2012 Dec;29(4):297-301. PMID: 23488213. |
| Oral health-related quality of life in Czech population | 2010 | Czech Republic/ EURO | Hodacová L, Smejkalová J, Cermáková E, Slezák R, Jacob V, Hlavácková E. Oral health-related quality of life in Czech population. Cent Eur J Public Health. 2010 Jun;18(2):76-80. doi: 10.21101/cejph.a3578. PMID: 20939256. |
| National Oral Health Survey of 3-Year-Old Children in Germany 2015/2016 | 2017 | Germany/ EURO | Basner R, Splietha CH, Santamariaa RM, Schülera E, Gablerb S, Schmoeckela J. National Oral Health Survey of 3-Year-Old Children in Germany 2015/2016. Caries Res 2017;51:290–385 |
| Epidemiological investigation of caries prevalence in first grade school children in Rhineland-Palatinate, Germany | 2015 | Germany/ EURO | Weusmann J, Mahmoodi B, Azaripour A, Kordsmeyer K, Walter C, Willershausen B. Epidemiological investigation of caries prevalence in first grade school children in Rhineland-Palatinate, Germany. Head Face Med. 2015 Oct 2;11:33. doi: 10.1186/s13005-015-0091-8. PMID: 26432570; PMCID: PMC4591732. |
| The Fifth German Oral Health Study (Fünfte Deutsche Mundgesundheitsstudie, DMS V) - rationale, design, and methods | 2014 | Germany/ EURO | Jordan RA, Bodechtel C, Hertrampf K, Hoffmann T, Kocher T, Nitschke I, Schiffner U, Stark H, Zimmer S, Micheelis W; DMS V Surveillance Investigators’ Group. The Fifth German Oral Health Study (Fünfte Deutsche Mundgesundheitsstudie, DMS V) - rationale, design, and methods. BMC Oral Health. 2014 Dec 29;14:161. doi: 10.1186/1472-6831-14-161. PMID: 25547464; PMCID: PMC4417261. |
| Caries prevalence in 12-year-old children from Germany. Results of the 2004 national survey | 2004 | Germany/ EURO | Schulte AG, Momeni A, Pieper K. Caries prevalence in 12-year-old children from Germany. Results of the 2004 national survey. Community Dent Health. 2006 Dec;23(4):197-202. PMID: 17194065. |
| The decline in dental caries among 12-year-old children in Germany between 1994 and 2000 | 2000 | Germany/ EURO | Pieper K, Schulte AG. The decline in dental caries among 12-year-old children in Germany between 1994 and 2000. Community Dent Health. 2004 Sep;21(3):199-206. PMID: 15470829. |
| Tand pleje information OPP | 2019 | Denmark/ EURO | https://www.tandplejeinformation.dk/wp-content/uploads/2020/01/SCOR-2019-standardtabeller.pdf |
| Dental health status and development trends among children and adolescents in Greenland | 2006 | Denmark/ EURO | Petersen PE, Christensen LB. Dental health status and development trends among children and adolescents in Greenland. Int J Circumpolar Health. 2006 Feb;65(1):35-44. doi: 10.3402/ijch.v65i1.17884. PMID: 16544646. |
| Findings from the oral health study of the Danish Health Examination Survey 2007-2008 | 2008 | Denmark/ EURO | Kongstad J, Ekstrand K, Qvist V, Christensen LB, Cortsen B, Grønbaek M, Holm-Pedersen P, Holmstrup P, Bardow A, Twetman S, Fiehn NE. Findings from the oral health study of the Danish Health Examination Survey 2007-2008. Acta Odontol Scand. 2013 Nov;71(6):1560-9. doi: 10.3109/00016357.2013.776701. Epub 2013 Apr 29. PMID: 23627881. |
| Indicadores de Riesgo Cariogénico en Adolescentes de Santo Domingo, República Dominicana | 2008 | Dominican Republic / OPAS | ollins J, Bobadilla M, Fresno MC. Indicadores de Riesgo Cariogénico en Adolescentes de Santo Domingo, República Dominicana. Revista Clínica de Periodoncia, Implantología y Rehabilitación Oral [Internet]. 2008 Nov 1 [cited 2022  Mar 18];1(3):86–9. |
| Prevalence and Risk Factors of Dental Caries among Preparatory School Children, Menoufia Governorate, Egypt | 2016 | Dominican Republic / OPAS | El, Hewida & Gabr, Hala. (2016). Prevalence and Risk Factors of Dental Caries among Preparatory School Children, Menoufia Governorate, Egypt. 1-11. |
| Assessing the prevalence of early childhood caries and the associated determinants in a group of preschool children: results from a national oral health survey in Egypt | 2019 | Egypt/ EMRO | Abou El Fadl, Reham & Fattah, Mona & Ezz, Mona. (2019). Assessing the prevalence of early childhood caries and the associated determinants in a group of preschool children: results from a national oral health survey in Egypt. Egyptian Dental Journal. 65. 31-39. 10.21608/edj.2015.71243. |
| Dental Caries Prevalence among a group of Egyptian Nurseries Children | 2011 | Egypt/ EMRO | <http://www.lifesciencesite.com/lsj/life0801/52_>  4652life0801_412_419.pdf |
| Prevalence of dental caries and associated factors among 12 years old students in Eritrea | 2017 | Eritrea/ AFRO | Andegiorgish, A. K., Weldemariam, B. W., Kifle, M. M., Mebrahtu, F. G., Zewde, H. K., Tewelde, M. G., ... & Tsegay, W. K. (2017). Prevalence of dental caries and associated factors among 12 years old students in Eritrea. BMC oral health, 17(1), 1-6. |
| Prevalence of dental caries and evaluation of mean DMFT index among secondary school students in Asmara, Eritrea | 2019 | Eritrea/ AFRO | Abdelhamid, Nada & Bahta, Habtom & Raja, Saud & Dhanni, Chandrakant & Hamida, Mohammed Elfatih. (2019). Prevalence of dental caries and evaluation of mean DMFT index among secondary school students in Asmara, Eritrea.. |
| Encuesta de Salud Oral en España 2020 | 2020 | Spain/ EURO | [https://dialnet.unirioja.es/servlet/articulo? codigo](https://dialnet.unirioja.es/servlet/articulo?%20%20codigo)=7772571 |
| Encuesta de Salud Oral en España 2010 | 2010 | Spain/ EURO | <https://dialnet.unirioja.es/servlet/articulo>?  codigo=6289439 |
| Encuesta de Salud Oral en España 2015 | 2015 | Spain/ EURO | https://diposit.ub.edu/dspace/bitstream/2445/103211/1/664717.pdf |
| Dental health of children and adolescents of Navarra, 2007 (4th edition) | 2009 | Spain/ EURO | Cortés Martinicorena FJ, Artázcoz J, Rosel E, González P, Asenjo MA, Sáinz de Murieta I, Bravo M. La salud dental de los niños y adolescentes de Navarra, 2007 (4 feminine edición) [Dental health of children and adolescents of Navarra, 2007 (4th edition)]. An Sist Sanit Navar. 2009 May-Aug;32(2):199-215. Spanish. doi: 10.23938/ASSN.0159. PMID: 19738644. |
| Encuesta de Salud Oral en España 2005 | 2005 | Spain/ EURO | https://scielo.isciii.es/pdf/rcoe/v11n4/encuesta.pdf |
| Prevalence of Dental Caries and Associated Factors Among Finote Selam Primary School Students Aged 12-20 years, Finote Selam Town, Ethiopia | 2016 | Ethiopia/ AFRO | Teshome, Amare & Gelaw, Asmare & Gizachew, Mucheye. (2016). Prevalence of Dental Caries and Associated Factors Among Finote Selam Primary School Students Aged 12-20 years, Finote Selam Town, Ethiopia. |
| Caries prevalence and use of dental services in Finnish children and Adolescents in 2009 | 2009 | Finland/ EURO | Wideström E, Järvinen S. Caries prevalence and use of dental services in Finnish children and Adolescents in 2009. OHDM. 2011; 10: 185-192. |
| Oral health in adults in France | 2006 | France/ EURO | Hescot P, Bourgeois D, Doury J. Oral health in adults in France. Int Dent J. 1997 Apr;47(2):94-9. doi: 10.1111/j.1875-595x.1997.tb00682.x. PMID: 9448793. |
| Etude epidemiologique de la carie dentaire en milieu scolaire a libreville, gabon. Epidemiological study of dental caries in school children in libreville, gabon | 2009 | Gabon/ AFRO | Koko, Jean & Ategbo, Simon & Ateba Ngoa, Ulysse & Moussavou, André. (2009). Etude epidemiologique de la carie dentaire en milieu scolaire a libreville, gabon. Epidemiological study of dental caries in school children in libreville, gabon. Clinics in Mother and Child Health. 6. |
| Report of the 2017 Detailed National Dental Inspection Programme of Primary 7 children and the Basic Inspection of Primary 1 and Primary 7 children | 2017 | United Kingdom/ EURO | National Dental Inspection Programme (NDIP). Scotland. 2017. Report of the 2017 Detailed National Dental Inspection Programme of Primary 7 children and the Basic Inspection of Primary 1 and Primary 7 children. |
| Feasibility, utility and impact of a national dental epidemiological survey of three-year-old children in England 2013 | 2013 | United Kingdom/ EURO | Davies GM, Neville J, Wilcox D. Feasibility, utility and impact of a national dental epidemiological survey of three-year-old children in England 2013. Community Dent Health. 2016 Jun;33(2):116-20. PMID: 27352465. |
| Report of the 2011 Detailed National Dental Inspection Programme | 2011 | United Kingdom/ EURO | https://ndip.scottishdental.org/wp-content/uploads/2014/07/ndip_scotland2011-P7.pdf |
| National Dental Inspection Programme (NDIP) 2013 | 2013 | United Kingdom/ EURO | https://ndip.scottishdental.org/wp-content/uploads/2021/11/ndip_scotland2013-P7.pdf |
| Children’s Dental Health Survey 2013 Technical Report England, Wales and Northern Ireland | 2015 | United Kingdom/ EURO | [https://doc.ukdataservice.ac.uk/doc/7774/mrdoc /pdf/ 7774_cdhs_2013_](https://doc.ukdataservice.ac.uk/doc/7774/mrdoc%20/pdf/%207774_cdhs_2013_)technical_report.pdf |
| National Dental Inspection Programme (NDIP) 2014 | 2014 | United Kingdom/ EURO | https://www.scottishdental.org/wp-content/uploads/2014/11/2014-10-28-NDIP-Report1.pdf |
| The caries experience of 11 to 12 year-old children in Scotland and Wales and 12 year-olds in England in 2008-2009: reports of co-ordinated surveys using BASCD methodology | 2009 | United Kingdom/ EURO | Davies GM, Jones CM, Monaghan N, Morgan MZ, Neville JS, Pitts NB. The caries experience of 11 to 12 year-old children in Scotland and Wales and 12 year-olds in England in 2008-2009: reports of co-ordinated surveys using BASCD methodology. Community Dent Health. 2012 Mar;29(1):8-13. PMID: 22482242. |
| The dental caries experience of 5-year-old children in England and Wales (2003/4) and in Scotland (2002/3). Surveys co-ordinated by the British Association for the Study of Community Dentistry | 2005 | United Kingdom/ EURO | Pitts NB, Boyles J, Nugent ZJ, Thomas N, Pine CM; British Association for the Study of Community Dentistry. The dental caries experience of 5-year-old children in England and Wales (2003/4) and in Scotland (2002/3). Surveys co-ordinated by the British Association for the Study of Community Dentistry. Community Dent Health. 2005 Mar;22(1):46-56. PMID: 15819117. |
| National Dental Inspection Programme (NDIP) 2017 | 2017 | United Kingdom/ EURO | https://www.scottishdental.org/wp-content/uploads/2017/10/ndip_scotland2017-P7.pdf |
| Nationwide 2.5-Year School-Based Public Health Intervention Program Designed to Reduce the Incidence of Caries in Children of Grenada | 2016 | Grenada/ OPAS | Wolff MS, Hill R, Wilson-Genderson M, Hirsch S, Dasanayake AP. Nationwide 2.5-Year School-Based Public Health Intervention Program Designed to Reduce the Incidence of Caries in Children of Grenada. Caries Res. 2016;50 Suppl 1:68-77. doi: 10.1159/000439058. Epub 2016 Apr 22. PMID: 27100682. |
| Dental caries among children in Georgia by age, gender, residence location and ethnic group | 2014 | Georgia/ EURO | Sgan-Cohen HD, Margvelashvili V, Bilder L, Kalandadze M, Gordon M, Margvelashvili M, Zini A. Dental caries among children in Georgia by age, gender, residence location and ethnic group. Community Dent Health. 2014 Sep;31(3):163-6. PMID: 25300151. |
| Survey of dental caries prevalence, dietary and oral hygiene habits among urban and rural 5 and 12-years old children in The Gambia | 2011 | Gambia/ AFRO | https://www.afrikaadats.com/wp-content/uploads/2021/04/us-barn-2003.pdf |
| National pathfinder survey of dental caries prevalence and treatment needs in The Gambia, the age group 35 - 44 years. | 2004 | Gambia/ AFRO | Blom, C., & Carlsson, M. National pathfinder survey of dental caries prevalence and treatment needs in The Gambia, the age group 35-44 years. |
| Caries prevalence of 5, 12 and 15-year-old Greek children: a national pathfinder survey | 2012 | Greece/ EURO | Oulis CJ, Tsinidou K, Vadiakas G, Mamai-Homata E, Polychronopoulou A, Athanasouli T. Caries prevalence of 5, 12 and 15-year-old Greek children: a national pathfinder survey. Community Dent Health. 2012 Mar;29(1):29-32. PMID: 22482246. |
| Caries prevalence and location and dental treatment needs in preschoolers in Athens--GENESIS project | 2007 | Greece/ EURO | Maragakis GM, Kapetanakou DN, Manios Y. Caries prevalence and location and dental treatment needs in preschoolers in Athens--GENESIS project. Community Dent Health. 2007 Dec;24(4):264-7. PMID: 18246846. |
| Dental caries prevalence and treatment needs of 5- to 12-year-old children in relation to area-based income and immigrant background in Greece | 2011 | Greece/ EURO | Gatou T, Koletsi Kounari H, Mamai-Homata E. Dental caries prevalence and treatment needs of 5- to 12-year-old children in relation to area-based income and immigrant background in Greece. Int Dent J. 2011 Jun;61(3):144-51. doi: 10.1111/j.1875-595X.2011.00031.x. PMID: 21692785; PMCID: PMC9374840. |
| Dental caries status and its associated factors among 5-year-old Hong Kong children: a cross-sectional study | 2017 | Hong Kong/ SEARO | Chen, K.J., Gao, S.S., Duangthip, D. et al. Dental caries status and its associated factors among 5-year-old Hong Kong children: a cross-sectional study. BMC Oral Health 17, 121 (2017). https://doi.org/10.1186/s12903-017-0413-2 |
| Oral health status and behaviours of preschool children in Hong Kong | 2012 | Hong Kong/ SEARO | Chu CH, Ho PL, Lo EC. Oral health status and behaviours of preschool children in Hong Kong. BMC Public Health. 2012 Sep 11;12(1):767. |
| Dental caries and erosion status of 12-year-old Hong Kong children | 2013 | Hong Kong/ SEARO | Zhang, S., Chau, A.M., Lo, E.C. et al. Dental caries and erosion status of 12-year-old Hong Kong children. BMC Public Health 14, 7 (2014). https://doi.org/10.1186/1471-2458-14-7 |
| Oral health survey 2011, Department of health | 2011 | Hong Kong/ SEARO | https://www.toothclub.gov.hk/en/en_ pdf/Oral_Health_Survey_2011/Oral_  Health_Survey_2011_WCAG_  20141112_(EN_Full).pdf |
| Risk indicators of oral health status among young adults aged 18 years analyzed by negative binomial regression | 2013 | Hong Kong/ SEARO | Lu, HX., Wong, M.C.M., Lo, E.C.M. et al. Risk indicators of oral health status among young adults aged 18 years analyzed by negative binomial regression. BMC Oral Health 13, 40 (2013). https://doi.org/10.1186/1472-6831-13-40 |
| Caries prevalence among schoolchildren in urban and rural Croatia | 2019 | Croatia/ EURO | Lešić S, Dukić W, Šapro Kriste Z, Tomičić V, Kadić S. Caries prevalence among schoolchildren in urban and rural Croatia. Cent Eur J Public Health. 2019 Sep;27(3):256-262. doi: 10.21101/cejph.a5314. PMID: 31580564. |
| Caries prevalence among schoolchildren in Zagreb, Croatia | 2011 | Croatia/ EURO | Dukić W, Delija B, Lulić Dukić O. Caries prevalence among schoolchildren in Zagreb, Croatia. Croat Med J. 2011 Dec 15;52(6):665-71. doi: 10.3325/cmj.2011.52.665. PMID: 22180264; PMCID: PMC3243318. |
| Presentation of DMFT/dmft Index in Croatia and Europe | 2015 | Croatia EURO | Radić M, Benjak T, Vukres VD, Rotim Ž, Zore IF. Presentation of DMFT/dmft Index in Croatia and Europe. Acta Stomatol Croat. 2015 Dec;49(4):275-84. doi: 10.15644/asc49/4/2. PMID: 27688411; PMCID: PMC4945336. |
| Incidence of caries in children of rural and subrural areas in Croatia | 2008 | Croatia/ EURO | Jurić H, Klarić T, Zagar M, Buković D Jr, Janković B, Spalj S. Incidence of caries in children of rural and subrural areas in Croatia. Coll Antropol. 2008 Mar;32(1):131-6. PMID: 18494198. |
| Dental caries experience in Croatian school children in Primorsko-Goranska county | 2013 | Croatia/ EURO | Jokić NI, Bakarcić D, Janković S, Malatestinić G, Dabo J, Majstorović M, Vuksan V. Dental caries experience in Croatian school children in Primorsko-Goranska county. Cent Eur J Public Health. 2013 Mar;21(1):39-42. doi: 10.21101/cejph.a3752. PMID: 23741899. |
| Changing Levels of Dental Caries over 30 Years among Children in a Country of Central and Eastern Europe - The Case of Hungary | 2020 | Hungary/ EURO | Szöke J, Petersen PE. Changing Levels of Dental Caries over 30 Years among Children in a ﻿Country of Central ﻿and Eastern Europe﻿ ﻿- ﻿The Case of ﻿﻿Hungary. Oral Health Prev Dent. 2020;18(1):177-183. doi: 10.3290/j.ohpd.a44322. PMID: 32238990. |
| Caries prevalence and tooth loss in Hungarian adult population: results of a national survey | 2008 | Hungary/ EURO | Madléna, M., Hermann, P., Jáhn, M. et al. Caries prevalence and tooth loss in Hungarian adult population: results of a national survey. BMC Public Health 8, 364 (2008). https://doi.org/10.1186/1471-2458-8-364 |
| Mothers' Dental Health Behaviors and Mother-Child's Dental Caries Experiences: Study of a Suburb Area in Indonesia | 2013 | Indonesia/ SEARO | Maharani, Diah & Rahardjo, Anton. (2013). Mothers' Dental Health Behaviors and Mother-Child's Dental Caries Experiences: Study of a Suburb Area in Indonesia. MAKARA of Health Series. 16. 10.7454/msk.v16i2.1632. |
| Dental and Gingival Status of 5 and 12-Year-Old Children in Jakarta and Its Satellite Cities | 2016 | Indonesia/ SEARO | Adiatman, M., Yuvana, A. L., Nasia, A. A., Rahardjo, A., Maharani, D. A., & Zhang, S. Dental and Gingival Status of 5 and 12-Year-Old Children in Jakarta and Its Satellite Cities. J Dent Indones. 2016;23(1): 5-9 |
| Indonesia: Epidemiological Profiles of Early Childhood Caries | 2019 | Indonesia/ SEARO | Amalia R, Chairunisa F, Alfian MF, Supartinah A. Indonesia: Epidemiological Profiles of Early Childhood Caries. Front Public Health. 2019 Aug 6;7:210. doi: 10.3389/fpubh.2019.00210. PMID: 31448251; PMCID: PMC6691044. |
| Does dental health of 6-year-olds reflect the reform of the Israeli dental care system? | 2016 | Israel/ EURO | Natapov, L., Sasson, A. & Zusman, S.P. Does dental health of 6-year-olds reflect the reform of the Israeli dental care system?. Isr J Health Policy Res 5, 26 (2016). https://doi.org/10.1186/s13584-016-0086-3 |
| Caries Prevalence Among Five-Year-Old Children Examined by the School Dental Service in Israel in 2007 | 2007 | Israel/ EURO | Natapov L, Gordon M, Pikovsky V, Kushnir D, Kooby E, Khoury G, Zusman SP. Caries Prevalence Among Five-Year-Old Children Examined by the School Dental Service in Israel in 2007. OHDMBSC. 2010; 9:25-31. |
| Epidemiology of dental caries among adolescents in Tamil Nadu, India | 2016 | India/ SEARO | Veerasamy A, Kirk R, Gage J. Epidemiology of dental caries among adolescents in Tamil Nadu, India. Int Dent J. 2016 Jun;66(3):169-77. doi: 10.1111/idj.12216. Epub 2016 Jan 29. PMID: 26825051; PMCID: PMC9376636. |
| Oral Health In India : A Report of the Multi centric Study | 2007 | India/ SEARO | Oral Health In India : A Report of the Multi centric Study. Ministry of Health and Family Welfare, Government of India and WHO collaborative Program, 2007. |
| Dental caries and associated factors in 12-year-old schoolchildren in Thiruvananthapuram, Kerala, India | 2005 | India/ SEARO | David J, Wang NJ, Astrøm AN, Kuriakose S. Dental caries and associated factors in 12-year-old schoolchildren in Thiruvananthapuram, Kerala, India. Int J Paediatr Dent. 2005 Nov;15(6):420-8. doi: 10.1111/j.1365-263X.2005.00665.x. PMID: 16238652. |
| Oral health status of 5 years and 12 years school going children in Chennai city--an epidemiological study | 2005 | India/ SEARO | Mahesh Kumar P, Joseph T, Varma RB, Jayanthi M. Oral health status of 5 years and 12 years school going children in Chennai city--an epidemiological study. J Indian Soc Pedod Prev Dent. 2005 Mar;23(1):17-22. doi: 10.4103/0970-4388.16021. PMID: 15858301. |
| Trends of Oral Diseases and Treatment Needs in 13-15 Year Old Students in Mo-sul City Center | 2013 | Iraq/ EMRO | Al-Sandook, Tahani & Al-Naimi, Raya & Jazrawi, Karam. (2013). Trends of Oral Diseases and Treatment Needs in 13-15 Year Old Students in Mo-sul City Center. Al-Rafidain Dental Journal. 13. 241-250. 10.33899/rden.2013.84797. |
| Dental caries in Iraqi 12-year-olds and background fluoride exposure | 2015 | Iraq/ EMRO | Matloob MH. Dental caries in Iraqi 12-year-olds and background fluoride exposure. Community Dent Health. 2015 Sep;32(3):163-9. PMID: 26513852. |
| Dental caries prevalence and risk factors among 12-year old schoolchildren from Baghdad, Iraq: a post-war survey | 2007 | Iraq/ EMRO | Ahmed NA, Astrøm AN, Skaug N, Petersen PE. Dental caries prevalence and risk factors among 12-year old schoolchildren from Baghdad, Iraq: a post-war survey. Int Dent J. 2007 Feb;57(1):36-44. doi: 10.1111/j.1875-595x.2007.tb00116.x. PMID: 17378348. |
| Dental caries experience and socio-economic status among Iranian children: a multilevel analysis | 2019 | Iran/ EMRO | hasemianpour, M., Bakhshandeh, S., Shirvani, A. et al. Dental caries experience and socio-economic status among Iranian children: a multilevel analysis. BMC Public Health 19, 1569 (2019). https://doi.org/10.1186/s12889-019-7693-1 |
| Prevalence of caries and fluorosis in adolescents in Iran | 2007 | Iran/ EMRO | Meyer-Lueckel H, Bitter K, Shirkhani B, Hopfenmuller W, Kielbassa AM. Prevalence of caries and fluorosis in adolescents in Iran. Quintessence Int. 2007 Jun;38(6):459-65. PMID: 17625628. |
| Oral health of Iranian children in 2004: a national pathfinder survey of dental caries and treatment needs | 2004 | Iran/ EMRO | Bayat-Movahed S, Samadzadeh H, Ziyarati L, Memary N, Khosravi R, Sadr-Eshkevari PS. Oral health of Iranian children in 2004: a national pathfinder survey of dental caries and treatment needs. East Mediterr Health J. 2011 Mar;17(3):243-9. PMID: 21735966. |
| Oral health and treatment needs among 15-year-olds in Tehran, Iran | 2008 | Iran/ EMRO | Yazdani R, Vehkalahti MM, Nouri M, Murtomaa H. Oral health and treatment needs among 15-year-olds in Tehran, Iran. Community Dent Health. 2008 Dec;25(4):221-5. PMID: 19149299. |
| Caries prevalence of permanent teeth: a national survey of children in Iceland using ICDAS | 2010 | Iceland/ EURO | Agustsdottir H, Gudmundsdottir H, Eggertsson H, Jonsson SH, Gudlaugsson JO, Saemundsson SR, Eliasson ST, Arnadottir IB, Holbrook WP. Caries prevalence of permanent teeth: a national survey of children in Iceland using ICDAS. Community Dent Oral Epidemiol. 2010 Aug;38(4):299-309. doi: 10.1111/j.1600-0528.2010.00538.x. Epub 2010 Apr 7. PMID: 20406275. |
| Prevalence of dental caries among schoolchildren from North-Eastern Italian population | 2018 | Italy/ EURO | Dobbiani A, Berton F, Perinetti G, Costantinides F, DI Lenarda R. Prevalence of dental caries among schoolchildren from North-Eastern Italian population. Minerva Stomatol. 2018 Apr;67(2):49-54. doi: 10.23736/S0026-4970.17.04041-9. Epub 2017 Dec 14. PMID: 29243446. |
| Survey of Caries Experience in 3- to 5-year-old Children in Northeast Italy in 2011 and Its Trend 1984-2011 | 2011 | Italy/ EURO | Ferro R, Besostri A, Olivieri A. Survey of Caries Experience in 3- to 5-year-old Children in Northeast Italy in 2011 and Its Trend 1984-2011. Oral Health Prev Dent. 2017;15(5):475-481. doi: 10.3290/j.ohpd.a38976. PMID: 28993822. |
| Relationship Between Social and Behavioural Factors and Caries Experience in Schoolchildren in Italy. | 2016 | Italy/ EURO | Ferrazzano GF, Sangianantoni G, Cantile T, Ingenito A. Relationship Between Social and Behavioural Factors and Caries Experience in Schoolchildren in Italy. Oral Health Prev Dent. 2016;14(1):55-61. doi: 10.3290/j.ohpd.a34996. PMID: 26525121. |
| Prevalence and severity of dental caries in 5- and 12-year old children in the Veneto Region (Italy). | 2007 | Italy/ EURO | Ferro R, Besostri A, Meneghetti B, Stellini E. Prevalence and severity of dental caries in 5- and 12-year old children in the Veneto Region (Italy). Community Dent Health. 2007 Jun;24(2):88-92. PMID: 17615823. |
| Caries prevalence and need for dental care in 13-18-year-olds in the Municipality of Milan, Italy | 2008 | Italy/ EURO | Campus G, Cagetti MG, Senna A, Sacco G, Strohmenger L, Petersen PE. Caries prevalence and need for dental care in 13-18-year-olds in the Municipality of Milan, Italy. Community Dent Health. 2008 Dec;25(4):237-42. PMID: 19149302. |
| Changing trend of caries from 1989 to 2004 among 12-year old Sardinian children | 2004 | Italy/ EURO | Campus, G., Sacco, G., Cagetti, M. et al. Changing trend of caries from 1989 to 2004 among 12-year old Sardinian children. BMC Public Health 7, 28 (2007). https://doi.org/10.1186/1471-2458-7-28 |
| Caries prevalence and tooth surface distribution in a group of 5-year-old Italian children | 2009 | Italy/ EURO | Ferro R, Besostri A, Olivieri A. Caries prevalence and tooth surface distribution in a group of 5-year-old Italian children. Eur Arch Paediatr Dent. 2009 Jan;10(1):33-7. doi: 10.1007/BF03262665. PMID: 19254525. |
| National Pathfinder survey of 12-year-old Children's Oral Health in Italy | 2007 | Italy/ EURO | Campus G, Solinas G, Cagetti MG, Senna A, Minelli L, Majori S, Montagna MT, Reali D, Castiglia P, Strohmenger L. National Pathfinder survey of 12-year-old Children's Oral Health in Italy. Caries Res. 2007;41(6):512-7. doi: 10.1159/000110884. Epub 2007 Nov 8. PMID: 17992014. |
| Risk/prevention indicators for the prevalence of dental caries in schoolchildren: results from the Italian OHSAR Survey | 2005 | Italy/ EURO | Perinetti G, Caputi S, Varvara G. Risk/prevention indicators for the prevalence of dental caries in schoolchildren: results from the Italian OHSAR Survey. Caries Res. 2005 Jan-Feb;39(1):9-19. doi: 10.1159/000081651. PMID: 15591729. |
| Dental plaque, caries prevalence and gingival conditions of 14-15-year-old schoolchildren in Jerash District, Jordan | 2006 | Jordan/ EMRO | El-Qaderi SS, Quteish Ta'ani D. Dental plaque, caries prevalence and gingival conditions of 14-15-year-old schoolchildren in Jerash District, Jordan. Int J Dent Hyg. 2006 Aug;4(3):150-3. doi: 10.1111/j.1601-5037.2006.00184.x. PMID: 16958744. |
| Oral health status among 6- and 12-year-old Jordanian schoolchildren | 2014 | Jordan/ EMRO | Rajab LD, Petersen PE, Baqain Z, Bakaeen G. Oral health status among 6- and 12-year-old Jordanian schoolchildren. Oral Health Prev Dent. 2014;12(2):99-107. doi: 10.3290/j.ohpd.a31220. PMID: 24624383. |
| Survey of Dental Diseases 2011. Ministry of Health and Welfare. 2012. | 2012 | Japan/ EMRO | <https://cappmediaprodst.blob.core.windows.net>  /media/2125/survey-of-dental-diseases-2011-japan.xls |
| National School Health Statistics | 2020 | Japan/ EMRO | <http://www.mext.go.jp/b_menu/toukei/chousa05>  /hoken/1268826.htm |
| National Oral Health Surveys. Medical Affairs Bureau. Ministry of Health and Welfare. 2005. | 2005 | Japan/ EMRO | https://www.health.gov.fj/wp-content/uploads/2018/03/Annual-Report-2005.pdf |
| Oral health status of children in rural schools in Kithoka, Kenya | 2014 | Kenya/ AFRO | Fujawa, DTyus, JCooper, JDzingle, JKapila, SEber, RGonzalez-Cabezas, CNdege, PKPeck, MPeck, SKapila, Y. Oral health status of children in rural schools in Kithoka, Kenya. Oral Health and Dental Management. 2014; 13(4):1174-1183. |
| Dental caries, gingivitis and the treatment needs among 12-year-olds | 2010 | Kenya/ AFRO | Owino RO, Masiga MA, Ng'ang'a PM, Macigo FG. Dental caries, gingivitis and the treatment needs among 12-year-olds. East Afr Med J. 2010 Jan;87(1):25-31. doi: 10.4314/eamj.v87i1.59950. PMID: 23057300. |
| Dental caries and oral health practices among 12 year old children in Nairobi West and Mathira West Districts, Kenya | 2012 | Kenya/ AFRO | Gathecha G, Makokha A, Wanzala P, Omolo J, Smith P. Dental caries and oral health practices among 12 year old children in Nairobi West and Mathira West Districts, Kenya. Pan Afr Med J. 2012;12:42. Epub 2012 Jun 22. Retraction in: Pan Afr Med J. 2015;22:233. PMID: 22891100; PMCID: PMC3415062. |
| Prevalence and pattern of early childhood caries among 3-5 year olds in Kiambaa, Kenya | 2010 | Kenya/ AFRO | Njoroge NW, Kemoli AM, Gatheche LW. Prevalence and pattern of early childhood caries among 3-5 year olds in Kiambaa, Kenya. East Afr Med J. 2010 Mar;87(3):134-7. doi: 10.4314/eamj.v87i3.62199. PMID: 23057310. |
| Oral Health Status among 12 Year Old Children in a Rural Kenyan Community | 2013 | Kenya/ AFRO | Fukuda, H., Ogada, C. N., Kihara, E., Wagaiyu, E. G., & Hayashi, Y. (2014). Oral health status among 12-yearold children in a rural Kenyan Community. |
| Kenya National Oral Health Survey Report 2015. Ministry of Health. Unit of Oral Health. Nairobi. Kenya | 2015 | Kenya/ AFRO | https://theperiodontist.co.ke/images/resources/KENYA-NATIONAL-ORAL-HEALTH-SURVEY-REPORT-2015.pdf |
| Dental caries status of Cambodian children and the effects of living environment factors | 2014 | Cambodia/ SEARO | Iwasaki, Hiroshi & Takanashi, Noboru & Nakayama, Akira & Iwata, Morimitsu & Maeda, Takahide & Miyazawa, Hiroo. (2014). Dental caries status of Cambodian children and the effects of living environment factors. Pediatric Dental Journal. 24. 10.1016/j.pdj.2014.07.001. |
| Oral health status and behaviours of children in rural districts of Cambodia | 2008 | Cambodia/ SEARO | Chu CH, Wong AW, Lo EC, Courtel F. Oral health status and behaviours of children in rural districts of Cambodia. Int Dent J. 2008 Feb;58(1):15-22. doi: 10.1111/j.1875-595x.2008.tb00172.x. PMID: 18350849. |
| Dental health status of 15 year-old schoolchildren in Comoros | 2013 | Comoros/ AFRO | Cisse D, Lo CM, Mohamed O, Diouf M, Faye D, Kane A, Ndiaye N. Etat de santé dentaire des élèves de 15 ans aux Comores [Dental health status of 15 year-old schoolchildren in Comoros]. Odontostomatol Trop. 2013 Sep;36(143):45-50. French. PMID: 24380121. |
| The decline in dental caries among children of different ages in Korea, 2000-2006 | 2006 | Korea, Repubic Of / SEARO | Han DH, Kim JB, Park DY. The decline in dental caries among children of different ages in Korea, 2000-2006. Int Dent J. 2010 Oct;60(5):329-35. PMID: 21141205. |
| Dental caries experience of Kuwaiti kindergarten schoolchildren | 2010 | Kwait/ EMRO | Al-Mutawa SA, Shyama M, Al-Duwairi Y, Soparkar P. Dental caries experience of Kuwaiti kindergarten schoolchildren. Community Dent Health. 2010 Dec;27(4):213-7. PMID: 21473355. |
| Prevalence and experience of dental caries among 12- and 15-year-old adolescents in Central Kazakhstan | 2017 | Kazakhstan/ EURO | Zharmagambetova A, Tuleutayeva S, Akhmetova S, Sumanova A, Baigulakov A, Sakenov T, Gorbatova MA, Grjibovski AM. Prevalence and experience of dental caries among 12- and 15-year-old adolescents in Central Kazakhstan. Public Health. 2017;151:118-120. |
| Oral health and the impact of socio-behavioural factors in a cross sectional survey of 12-year old school children in Laos | 2009 | Lao People's Democratic Republic/ WPRO | Jürgensen N, Petersen PE. Oral health and the impact of socio-behavioural factors in a cross sectional survey of 12-year old school children in Laos. BMC Oral Health. 2009 Nov 16;9:29. doi: 10.1186/1472-6831-9-29. PMID: 19917089; PMCID: PMC2781791. |
| International Perspectives and Practical Applications on Fluorides and Fluoridation. | 2004 | Saint Lucia/ OPAS | Saskla Estupiñán‐Day. International Perspectives and Practical Applications on Fluorides and Fluoridation. Journal of Public Health Dentistry. 2004 Sep 1;64(s1):40–3 |
| National Oral Health Survey Sri Lanka 2015-2016. Colombo: Ministry of Health, Nutrition and Indigenous Medicine (Sri Lanka) | 2016 | Sri Lanka/ SEARO | https://www.health.gov.lk/wp-content/uploads/2028/Oral-Health-Report-cover-combined.pdf |
| Prevalence and severity of dental caries among 18-year-old Lithuanian adolescents | 2016 | Lithuania/ EURO | Žemaitienė M, Grigalauskienė R, Vasiliauskienė I, Saldūnaitė K, Razmienė J, Slabšinskienė E. Prevalence and severity of dental caries among 18-year-old Lithuanian adolescents. Medicina (Kaunas). 2016;52(1):54-60. doi: 10.1016/j.medici.2016.01.006. Epub 2016 Jan 29. PMID: 26987501. |
| Analysis of dental caries prevention program in 7-12-year-old Lithuanian schoolchildren | 2009 | Lithuania/ EURO | Saldūnaite K, Pūriene A, Milciuviene S, Brukiene V, Kutkauskiene J. 7-12 metu Lietuvos moksleiviu krūminiu dantu eduonies profilaktikos programos analize [Analysis of dental caries prevention program in 7-12-year-old Lithuanian schoolchildren]. Medicina (Kaunas). 2009;45(11):887-95. Lithuanian. PMID: 20051721. |
| Dental caries prevalence among 12-15-year-olds in Lithuania between 1983 and 2005 | 2005 | Lithuania/ EURO | Milciuviene S, Bendoraitiene E, Andruskeviciene V, Narbutaite J, Sakalauskiene J, Vasiliauskiene I, Slabsinskiene E. Dental caries prevalence among 12-15-year-olds in Lithuania between 1983 and 2005. Medicina (Kaunas). 2009;45(1):68-76. PMID: 19223708. |
| Caries Prevalence and Severity for 12-Year-Old Children in Latvia | 2017 | Latvia/ EURO | Maldupa I. Uribeb S. Nizamovsa M. Senakolaa E. Caries Prevalence and Risk Factors Among 12-Year-Old Latvian Schoolchildren. Caries Res 2017;51:290–385. (Abstracts: 64th ORCA Congress) |
| Dental Caries Experience and Oral Health Practice among 12-Years-Old Schoolchildren | 2022 | Libyan Arab Jamahiriya/ EMRO | Mansur, Eman & a Ayyad, Halima & Elzahaf, Raga. (2022). Dental Caries Experience and Oral Health Practice among 12-Years-Old Schoolchildren. International Journal of Clinical Preventive Dentistry. 18. 1-7. 10.15236/ijcpd.2022.18.1.1. |
| Prevalence and severity of dental caries in Libyan schoolchildren | 2011 | Libyan Arab Jamahiriya/ EMRO | Huew R, Waterhouse PJ, Moynihan PJ, Maguire A. Prevalence and severity of dental caries in Libyan schoolchildren. Int Dent J. 2011 Aug;61(4):217-23. doi: 10.1111/j.1875-595X.2011.00060.x. PMID: 21851354; PMCID: PMC9374798. |
| The state of oral health in children at the age of 12 in Montenegro | 2011 | Montenegro/ EURO | Djuricković M, Ivanović M. [The state of oral health in children at the age of 12 in Montenegro]. Vojnosanit Pregl. 2011 Jul;68(7):550-5. Serbian. doi: 10.2298/vsp1107550d. PMID: 21899174. |
| Dental Caries Experience among 15-years Old Children in the Southeast Region of the Republic of Macedonia | 2015 | Republic of Macedonia/ EURO | Ambarkova, Vesna & Panova, Olgica. (2015). Dental Caries Experience among 15-years Old Children in the Southeast Region of the Republic of Macedonia. Oral health and dental management. 14. 366-373. |
| Dental caries experience among primary school children in the Eastern Region of the Republic of Macedonia | 2014 | Republic of Macedonia/ EURO | Ambarkova V, Ivanova V. Dental caries experience among primary school children in the Eastern Region of the Republic of Macedonia. Oral Health Dent Manag. 2014 Mar;13(1):1-7. PMID: 24603908. |
| Oral health care provision systems in the black sea countries part 14: the Republic of Macedonia | 2013 | Republic of Macedonia/ EURO | Nikolovska J. Oral health care provision systems in the black sea countries part 14: the Republic of Macedonia. Oral Health Dent Manag. 2013 Jun;12(2):61-4. PMID: 23756420. |
| Myanmar National Oral Health Survey | 2009 | Myanmar/ SEARO | https://cdn.who.int/media/docs/default-source/country-profiles/oral-health/oral-health-mmr-2022-country-profile.pdf?sfvrsn=4bd0639e_6 |
| Early childhood caries and related risk factors in Mongolian children | 2009 | Mongolia/ WPRO | Jigjid B, Ueno M, Shinada K, Kawaguchi Y. Early childhood caries and related risk factors in Mongolian children. Community Dent Health. 2009 Jun;26(2):121-8. PMID: 19626745. |
| The Impact of Changing Fluoride Concentrations in the Water Supplies in the Maltese Islands on Caries Prevalence in 12-Year-Old Maltese Schoolchildren | 2010 | Malta/ EURO | Vassallo P. The impact of changing fluoride concentrations in the water supplies in the Maltese Islands on caries prevalence in 12-year-old Maltese schoolchildren. OHDMBSC. 2010; 9; 235-243. |
| Magnitude of dental caries, missing and filled teeth in Malawi: National Oral Health Survey | 2016 | Malawi/ AFRO | Msyamboza KP, Phale E, Namalika JM, Mwase Y, Samonte GC, Kajirime D, Sumani S, Chalila PD, Potani R, Mwale GC, Kathyola D, Mukiwa W. Magnitude of dental caries, missing and filled teeth in Malawi: National Oral Health Survey. BMC Oral Health. 2016 Mar 9;16:29. doi: 10.1186/s12903-016-0190-3. PMID: 26956884; PMCID: PMC4784360. |
| Resultados del Sistema de Vigilancia Epidemiológica de Patologías Bucales SIVEPAB 2018 | 2018 | Mexico/OPAS | https://www.gob.mx/cms/uploads/attachment /file/525756/20200116_archivo_SIVEPAB-18_1nov19_1_.pdf |
| Informe de Caries Dental, Encuesta Nacional de Caries y Fluorosis Dental 2011-2014 | 2014 | Mexico/OPAS | https://www.gob.mx/cms/uploads/attachment/file  /422450/Informe_de_Caries_Dental__Encuesta_  Nacional_de_Caries_y_Fluorosis_Dental_2011-2014_1.pdf |
| Estudio epidemiológico de caries dental  en escolares del estado de Baja California,  México, 2010 | 2010 | Mexico/OPAS | Verdugo Díaz, RDJ, Llodra Calvo JC, Sánchez-Rubio Carrillo RM, Barreras Serrano A, Sánchez-Rubio Carrillo RA, Torres Arellano M E, Gómez-Llanos Juárez, H. Estudio epidemiológico de caries dental en escolares del estado de Baja California, México, 2010/2010 Epidemiological Study of Dental Caries in Schoolchildren of the Baja California State, Mexico. Universitas Odontologica, 2013; 32: 99-108 |
| National Health and Morbidity Survey 2017: National Oral Health Survey of Schoolchildren 2017 | 2017 | Malaysia/ WPRO | <https://hq.moh.gov.my/ohd/images/pdf/research>  /NHMS%202017%20NOHSS%202017%20  Vol%20II%20Oral%20Health%20Status%20  of%2012%20yr%20olds.pdf |
| Oral Health Division, Ministry of Health Malaysia. National Oral Health Survey of Preschool Children 2015 | 2015 | Malaysia/ WPRO | <https://hq.moh.gov.my/ohd/images/pdf/compendium>  /NOHPS-2015-CARIES.pdf |
| Oral Health Division, Ministry of Health, Malaysia. National Oral Health Survey of School Children 2006 | 2006 | Malaysia/ WPRO | https://hq.moh.gov.my/ohd/images/pdf/xtvtnsop/Oral-Healthcare-for-Schoolchildren-in-Malaysia-2006.pdf |
| National Oral Health Survey of School Children 2007 | 2007 | Malaysia/ WPRO | National Oral Health Survey of School Children 2007 (NOHSS 2007). Oral Health Division. Ministry of Health. Malaysia. |
| Dental caries in adolescents from public schools in Maputo, Mozambique | 2010 | Mozambique/ AFRO | Mapengo MA, Marsicano JA, Garcia de Moura P, Sales-Peres A, Hobdell M, de Carvalho Sales-Peres SH. Dental caries in adolescents from public schools in Maputo, Mozambique. Int Dent J. 2010 Aug;60(4):273-81. PMID: 20949758. |
| Dental caries and periodontal diseases in Mozambique | 2019 | Mozambique/ AFRO | DOMINGOS, M. A. A. M.; MEPATIA, A. I. .; XAVIER, C. N. H. .; BARRIE, R. B. .; NAIDOO, S. .; MARSICANO, J. A. .; SALES PERES, S. H. de C. . Dental caries and periodontal diseases in Mozambique. Research, Society and Development, [S. l.], v. 11, n. 1, p. e46511125221, 2022. DOI: 10.33448/rsd-v11i1.25221. Disponível em: https://rsdjournal.org/index.php/rsd/article/view/25221. Acesso em: 28 feb. 2024. |
| National oral health survey Namibia 2010/11 | 2010 | Namibia/ AFRO | <https://indexmedicus.afro.who.int/iah/fulltext/>  National_oral_health_survey_Namibia.pdf |
| Common oral conditions and correlates: an oral health survey in Kwara State Nigeria | 2017 | Nigeria/ AFRO | Tobin, A.O., Ajayi, I.O. Common oral conditions and correlates: an oral health survey in Kwara State Nigeria. BMC Res Notes 10, 568 (2017). https://doi.org/10.1186/s13104-017-2894-0 |
| Impact of dental caries and its treatment on the quality of life of 12- to 15-year-old adolescents in Benin, Nigeria | 2016 | Nigeria/ AFRO | Chukwumah NM, Folayan MO, Oziegbe EO, Umweni AA. Impact of dental caries and its treatment on the quality of life of 12- to 15-year-old adolescents in Benin, Nigeria. Int J Paediatr Dent. 2016 Jan;26(1):66-76. doi: 10.1111/ipd.12162. Epub 2015 Apr 11. PMID: 25864531. |
| Prevalence and severity of dental caries among pensioners in Benin City, Nigeria | 2009 | Nigeria/ AFRO | Okeigbemen SA, Jeboda SO, Umweni AA. Prevalence and severity of dental caries among pensioners in Benin City, Nigeria. Odontostomatol Trop. 2009 Mar;32(125):11-6. PMID: 19711836. |
| Changes in the prevalence of dental caries in primary school children in Lagos State, Nigeria | 2014 | Nigeria/ AFRO | Sofola OO, Folayan MO, Oginni AB. Changes in the prevalence of dental caries in primary school children in Lagos State, Nigeria. Niger J Clin Pract. 2014 Mar-Apr;17(2):127-33. doi: 10.4103/1119-3077.127419. PMID: 24553018. |
| Oral health status of 12-year-old Nigerian children | 2006 | Nigeria/ AFRO | Agbelusi GA, Jeboda SO. Oral health status of 12-year-old Nigerian children. West Afr J Med. 2006 Jul-Sep;25(3):195-8. doi: 10.4314/wajm.v25i3.28277. PMID: 17191418. |
| Gebit Fit, een onderzoek naar de mondgezondheid en het tandheelkundig preventief gedrag van volwassenen in Nederland in 2013 | 2013 | Netherlands/ EURO | Schuller AA, I. van Kempen, E. Vermaire, J. Poorterman, Verlinden A, Hofstetter H, et al. Gebit fit : een onderzoek naar de mondgezondheid en het tandheelkundig preventief gedrag van volwassenen in Nederland in 2013. 2014 Jan 1; |
| Mondgezondheid volwassenen 2007 | 2007 | Netherlands/ EURO | https://www.tno.nl/media/1929/rap  portmondgezondheid_2007.pdf |
| Analysis of the ups and downs of caries experience among Norwegian children aged five years between 1997 and 2003 | 2003 | Norway EURO | Haugejorden O, Birkeland JM. Analysis of the ups and downs of caries experience among Norwegian children aged five years between 1997 and 2003. Acta Odontol Scand. 2005 Apr;63(2):115-22. doi: 10.1080/00016350510019784. PMID: 16134551. |
| Oral Health Condition of School Children in Nawalparasi District, Nepal | 2015 | Nepal/ SEARO | Thapa P, Aryal KK, Dhimal M, Mehata S, Pokhrel AU, Pandit A, Pandey AR, Bista B, Dhakal P, Karki KB, Pradhan S. Oral Health Condition of School Children in Nawalparasi District, Nepal. J Nepal Health Res Counc. 2015 Jan-Apr;13(29):7-13. PMID: 26411706. |
| Oral health status of 5 years and 12 years old school going children in rural Gurgaon, India: an epidemiological study | 2014 | New Zealand/ WPRO | Mittal M, Chaudhary P, Chopra R, Khattar V. Oral health status of 5 years and 12 years old school going children in rural Gurgaon, India: an epidemiological study. J Indian Soc Pedod Prev Dent. 2014 Jan-Mar;32(1):3-8. doi: 10.4103/0970-4388.127039. PMID: 24531594. |
| New Zealand Oral Health Survey 2009 | 2009 | New Zealand/ WPRO | https://www.health.govt.nz/nz-health-statistics/health-statistics-and-data-sets/oral-health-data-and-stats/age-5-and-year-8-oral-health-data-community-oral-health-service |
| Ministry of Health - Dental health status of 5 and 12 years old children 2011 | 2011 | New Zealand/ WPRO | https://www.health.govt.nz/nz-health-statistics/health-statistics-and-data-sets/oral-health-data-and-stats/age-5-and-year-8-oral-health-data-community-oral-health-service |
| Ministry of Health - (Dental health status of 5 and 12 years old children 2004 | 2004 | New Zealand/ WPRO | https://www.health.govt.nz/nz-health-statistics/health-statistics-and-data-sets/oral-health-data-and-stats/age-5-and-year-8-oral-health-data-community-oral-health-service |
| Ministry of Health - (Dental health status of 5 and 12 years old children 2005 | 2005 | New Zealand/ WPRO | https://www.health.govt.nz/nz-health-statistics/health-statistics-and-data-sets/oral-health-data-and-stats/age-5-and-year-8-oral-health-data-community-oral-health-service |
| Ministry of Health - (Dental health status of 5 and 12 years old children 2007 | 2007 | New Zealand/ WPRO | https://www.health.govt.nz/nz-health-statistics/health-statistics-and-data-sets/oral-health-data-and-stats/age-5-and-year-8-oral-health-data-community-oral-health-service |
| Oman National oral health survey for the 12 &15 years old 2006 | 2006 | Oman/ EMRO | Oman National oral health survey for the 12 &15 years old 2006. Dental & Oral Health department. Ministry of Health. courtesy of - Dr Salahudeen Al Bulushi. Director Dental Health Affairs |
| National Monitoring and Evaluation Dental Survey (NMEDS), 2011 | 2011 | Philippines/ WPRO | <https://vle.upm.edu.ph/pluginfile.php/145950/>  mod_folder/content/0/Readings%20and%20o  ther%20Resources/2018%20National%20Sur  vey%20on%20Oral%20Health%20in%20the%2  0Philippines.pdf |
| Urgent oral health needs of Filipino children: The results of the 2006 national oral health survey | 2006 | Philippines/ WPRO | Monse B. Yanga-Mabunga. Urgent oral health needs of Filipino children: The results of the 2006 national oral health survey. Develop Dent 2007;8:7-9. |
| Prevalence and factors related to dental caries among pre-school children of Saddar town, Karachi, Pakistan: a cross-sectional study | 2012 | Pakistan/ EMRO | Dawani, N., Nisar, N., Khan, N. et al. Prevalence and factors related to dental caries among pre-school children of Saddar town, Karachi, Pakistan: a cross-sectional study. BMC Oral Health 12, 59 (2012). https://doi.org/10.1186/1472-6831-12-59 |
| Dental caries experience in preschool children: is it related to a child's place of residence and family income? | 2011 | Pakistan/ EMRO | Sufia S, Chaudhry S, Izhar F, Syed A, Mirza BA, Khan AA. Dental caries experience in preschool children: is it related to a child's place of residence and family income? Oral Health Prev Dent. 2011;9(4):375-9. PMID: 22238736. |
| National Epidemiological Survey 2011. MInistry of Health. Personal communication - Dr Elżbieta Małkiewicz. 2012 | 2012 | Poland/ EURO | <http://www.przeglepidemiol.pzh.gov.pl/files/peissues/>  PE_nr_4_2017_srodek_calosc_net.pdf |
| Dental Caries Level and Sugar Consumption in 12-Year-Old Children from Poland | 2016 | Poland/ EURO | Olczak-Kowalczyk D, Turska A, Gozdowski D, Kaczmarek U. Dental Caries Level and Sugar Consumption in 12-Year-Old Children from Poland. Adv Clin Exp Med. 2016 May-Jun;25(3):545-50. doi: 10.17219/acem/61615. PMID: 27629744. |
| Dental caries among 12-year-old children in northern Poland between 1987 and 2003 | 2003 | Poland/ EURO | Emerich K, Adamowicz-Klepalska B. Dental caries among 12-year-old children in northern Poland between 1987 and 2003. Eur J Paediatr Dent. 2007 Sep;8(3):125-30. PMID: 17919060. |
| Trends in dental caries experience among children and adolescents in northern Poland between 1995 and 2003 | 2005 | Poland/ EURO | Emerich K, Adamowicz-Klepalska B. Trends in dental caries experience among children and adolescents in northern Poland between 1995 and 2003. Community Dent Health. 2010 Dec;27(4):218-21. PMID: 21473356. |
| Persistent oral health disparity in 12-year-old Hispanics: a cross-sectional study | 2016 | Puerto Rico/ OPAS | Elias-Boneta AR, Toro MJ, Rivas-Tumanyan S, Murillo M, Orraca L, Encarnacion A, Cernigliaro D, Toro-Vizcarrondo C, Psoter WJ. Persistent oral health disparity in 12-year-old Hispanics: a cross-sectional study. BMC Oral Health. 2016 Feb 1;16:10. doi: 10.1186/s12903-016-0162-7. PMID: 26830842; PMCID: PMC4736133. |
| Health-Promoting Schools Project for Palestine Children's Oral Health | 2023 | Palestinian Territory, Occupied/ EMRO | Abuhaloob L, Petersen PE. Health-Promoting Schools Project for Palestine Children's Oral Health. Int Dent J. 2023 Oct;73(5):746-753. doi: 10.1016/j.identj.2023.03.011. Epub 2023 Apr 27. PMID: 37120392; PMCID: PMC10509424. |
| Prevalence of dental caries and fissure sealants in a Portuguese sample of adolescents | 2015 | Portugal/ EURO | Veiga, N. J., Pereira, C. M., Ferreira, P. C., & Correia, I. J. (2015). Prevalence of dental caries and fissure sealants in a Portuguese sample of adolescents. PloS one, 10(3), e0121299. |
| Caries prevalence and treatment needs in young people in Portugal: the third national study | 2017 | Portugal/ EURO | Calado, R., Ferreira, C. S., Nogueira, P., & Melo, P. (2017). Caries prevalence and treatment needs in young people in Portugal: the third national study. Community Dent Health, 34(2), 107-11. |
| Trends on the Prevalence and Severity of Dental Caries in Portuguese Children and Teenagers | 2016 | Portugal/ EURO | Melo P. Ferreira CS. Nogueira P. Calado R. Trends on the Prevalence and Severity of Dental Caries in Portuguese Children and Teenagers. Caries Res 2016;50:201. (Abstracts- 63rd ORCA Congress) and personal communication with Dr Melo. |
| Oral health status of six-year-old children in Qatar: findings from the national oral health survey | 2016 | Qatar/ EMRO | Al-Thani M, Al-Thani A, Al-Emadi A, Al-Chetachi W, Akram H, Poovelil B. Oral health status of six-year-old children in Qatar: findings from the national oral health survey. International Journal of Dental Hygiene. 2016 Nov 20;16(2):225–32. |
| Oral Health Status of 12- and 15-Year-Old Students in Qatar: Findings From the National Oral Health Survey | 2018 | Qatar/ EMRO | Al-Thani, Mohammed & Al-Thani, Al-Anoud & Al-Emadi, Abdulla & Al-Chetachi, Walaa & Vinodson, Benjamin & Akram, Hammad. (2018). Oral Health Status of 12- and 15-Year-Old Students in Qatar: Findings From the National Oral Health Survey. International Journal of Basic Science in Medicine. 3. 32-37. 10.15171/ijbsm.2018.06. |
| Caries experience among Romanian schoolchildren: prevalence and trends 1992-2011 | 2011 | Romanian/ EURO | Baciu D, Danila I, Balcos C, Gallagher JE, Bernabé E. Caries experience among Romanian schoolchildren: prevalence and trends 1992-2011. Community Dent Health. 2015 Jun;32(2):93-7. PMID: 26263602. |
| Prevalence and Severity of Dental Caries in 6- and 12-Year-Old Children in Constanta District (Urban Area | 2009 | Romanian/ EURO | Nuca C. Amariei C. Borutta A. Petcu L. Prevalence and Severity of Dental Caries in 6- and 12-Year-Old Children in Constanta District (Urban Area). Romania. OHDMBSC. 2009;8:19-24. |
| Caries experience in schoolchildren in Bucharest, Romania: the PAROGIM study | 2014 | Romanian/ EURO | Funieru C, Twetman S, Funieru E, Dumitrache AM, Sfeatcu RI, Baicus C. Caries experience in schoolchildren in Bucharest, Romania: the PAROGIM study. J Public Health Dent. 2014 Spring;74(2):153-8. doi: 10.1111/jphd.12039. Epub 2013 Oct 9. PMID: 24308765. |
| Oral Health in 12- and 15-Year-Old Children in Serbia: A National Pathfinder Study. Int J Environ Res Public Health | 2022 | Serbia/ EURO | Peric T, Campus G, Markovic E, Petrovic B, Soldatovic I, Vukovic A, Kilibarda B, Vulovic J, Markovic J, Markovic D. Oral Health in 12- and 15-Year-Old Children in Serbia: A National Pathfinder Study. Int J Environ Res Public Health. 2022 Sep 27;19(19):12269. doi: 10.3390/ijerph191912269. PMID: 36231568; PMCID: PMC9566810. |
| Government of Serbia, Ministry of Health. National Program for Prevention of Oral Diseases in Republic of Serbia. Službeni Glasnik RS 22/2009 | 2022 | Serbia/ EURO | Government of Serbia, Ministry of Health National  Program for Prevention of Oral Diseases  in Republic of Serbia. Službeni Glasnik  RS 22/2009. [(accessed on 17 June 2017)].  Available online: <http://demo.paragraf.rs/>  demo/combined/Old/t/t2009_04/t04_0044  .htm (In Serbian) |
| Changes in Dental Caries in Preschool Children in Northwest Russia between 2007 and 2016 | 2007 | Russian Federation/ EURO | https://karger.com/cre/article-pdf/51/4/290/2503010/000471777.pdf |
| Provision of Oral Health Care in the Black Sea Countries Part 5: The Russian Federation | 2010 | Russian Federation/ EURO | OO, Janushevich & Fabrikant, Kate & AS, Kazakov. (2010). Provision of Oral Health Care in the Black Sea Countries Part 5: The Russian Federation.. OHDMBSC. 9. 59-62. |
| Building oral health research infrastructure: the first national oral health survey of Rwanda | 2018 | Rwanda/ AFRO | Morgan JP, Isyagi M, Ntaganira J, Gatarayiha A, Pagni SE, Roomian TC, Finkelman M, Steffensen JEM, Barrow JR, Mumena CH, Hackley DM. Building oral health research infrastructure: the first national oral health survey of Rwanda. Glob Health Action. 2018;11(1):1477249. doi: 10.1080/16549716.2018.1477249. PMID: 29860930; PMCID: PMC5990941. |
| Oral health status of 12-year-old male schoolchildren in Medina, Saudi Arabia | 2014 | Saudi Arabia/ EMRO | Bhayat A, Ahmad MS. Oral health status of 12-year-old male schoolchildren in Medina, Saudi Arabia. East Mediterr Health J. 2014 Dec 17;20(11):732-7. PMID: 25601812. |
| Prevalence of dental caries in primary and permanent teeth and its relation with tooth brushing habits among schoolchildren in Eastern Saudi Arabia | 2015 | Saudi Arabia/ EMRO | Farooqi FA, Khabeer A, Moheet IA, Khan SQ, Farooq I, ArRejaie AS. Prevalence of dental caries in primary and permanent teeth and its relation with tooth brushing habits among schoolchildren in Eastern Saudi Arabia‬‬‬‬‬‬‬‬. Saudi Med J. 2015 Jun;36(6):737-42. doi: 10.15537/smj.2015.6.10888. PMID: 25987118; PMCID: PMC4454910. |
| Caries prevalence, severity, and pattern in preschool children | 2008 | Saudi Arabia/ EMRO | Wyne AH. Caries prevalence, severity, and pattern in preschool children. J Contemp Dent Pract. 2008 Mar 1;9(3):24-31. PMID: 18335116. |
| Oral health survey of school children aged 6 and 12 years in the Solomon Islands | 2007 | Solomon Island/ WPRO | Vane E S. Oral health survey of school children aged 6 and 12 years in the Solomon Islands.Thesis. Faculty of Dentistry.Sydney. Australia. 2007. |
| Oral health status of children in the Republic of Seychelles 2005 | 2000 | Seychelles/ SEARO | Ernesta K, Dogley M, Tillberg A. Oral health status of children in the Republic of Seychelles (2005). Ministry of Health - Dental Services, 2007. |
| A survey of oral health in a Sudanese population | 2012 | Sudan/ EMRO | Khalifa, N., Allen, P.F., Abu-bakr, N.H. et al. A survey of oral health in a Sudanese population. BMC Oral Health 12, 5 (2012). https://doi.org/10.1186/1472-6831-12-5 |
| Prevalence of dental caries and toothbrushing habits among preschool children in Khartoum State, Sudan | 2016 | Sudan/ EMRO | Elidrissi SM, Naidoo S. Prevalence of dental caries and toothbrushing habits among preschool children in Khartoum State, Sudan. Int Dent J. 2016 Aug;66(4):215-20. doi: 10.1111/idj.12223. Epub 2016 Apr 8. PMID: 27061284; PMCID: PMC9376632. |
| Oral health status of 12-year-old school children in Khartoum state, the Sudan; a school-based survey | 2009 | Sudan/ EMRO | Nurelhuda, N.M., Trovik, T.A., Ali, R.W. et al. Oral health status of 12-year-old school children in Khartoum state, the Sudan; a school-based survey. BMC Oral Health 9, 15 (2009). https://doi.org/10.1186/1472-6831-9-15 |
| Quality Registry for Caries and Periodontal Diseases - a framework for quality development in dentistry | 2019 | Sweden/ EURO | von Bültzingslöwen I, Östholm H, Gahnberg L, Ericson D, Wennström JL, Paulander J. Swedish Quality Registry for Caries and Periodontal Diseases - a framework for quality development in dentistry. Int Dent J. 2019 Oct;69(5):361-368. doi: 10.1111/idj.12481. Epub 2019 Apr 18. PMID: 31001827; PMCID: PMC6790561. |
| Oral health in the adult population of Skåne, Sweden: a clinical study. Acta Odontol Scand | 2012 | Sweden/ EURO | Lundegren N, Axtelius B, Akerman S. Oral health in the adult population of Skåne, Sweden: a clinical study. Acta Odontol Scand. 2012 Dec;70(6):511-9. doi: 10.3109/00016357.2011.640279. Epub 2011 Dec 20. PMID: 22181829. |
| Dental caries prevalence and distribution among preschoolers in Singapore | 2009 | Singapore/ WPRO | Gao XL, Hsu CY, Loh T, Koh D, Hwamg HB, Xu Y. Dental caries prevalence and distribution among preschoolers in Singapore. Community Dent Health. 2009 Mar;26(1):12-7. PMID: 19385434. |
| Epidemiology of Caries in 12-Year-Olds in Slovenia | 2013 | Slovenia/ EURO | Vrbič V, Vrbič M. Epidemiology of Caries in 12-Year-Olds in Slovenia 1987-2013. Oral Health Prev Dent. 2016;14(5):467-473. doi: 10.3290/j.ohpd.a36466. PMID: 27351731. |
| Unmet need in Sierra Leone: a national oral health survey of schoolchildren | 2022 | Sierra Leone/ AFRO | Ghotane SG, Challacombe SJ, Don-Davis P, Kamara D, Gallagher JE. Unmet need in Sierra Leone: a national oral health survey of schoolchildren. BDJ Open. 2022 Jun 14;8(1):16. doi: 10.1038/s41405-022-00107-7. PMID: 35701398; PMCID: PMC9194785. |
| Prevalence of dental caries among 12-year old schoolchildren in the Dakar region | 2014 | Senegal/ AFRO | Benoist FL, Bane K, Aidara AW, Ndiaye D, Chouker Y, Kane AW. Prévalence de la carie dentaire chez les élèves de 12 ans de la région de Dakar [Prevalence of dental caries among 12-year old schoolchildren in the Dakar region]. Odontostomatol Trop. 2014 Jun;37(146):58-64. French. PMID: 25223148. |
| Prevalence of dental caries in the first permanent molar and associated risk factors among sixth-grade students in São Tomé Island | 2021 | Sao Tome and Principe/ AFRO | Que L, Jia M, You Z, Jiang LC, Yang CG, Quaresma AAD, das Neves EMAA. Prevalence of dental caries in the first permanent molar and associated risk factors among sixth-grade students in São Tomé Island. BMC Oral Health. 2021 Sep 28;21(1):483. doi: 10.1186/s12903-021-01846-z. PMID: 34583665; PMCID: PMC8479893. |
| Dental caries prevalence and severity in a children population of S. Tomé | 2013 | Sao Tome and Principe/ AFRO | Coimbra F, Mendes S, Bernardo M. Dental caries prevalence and severity in a children population of S. Tomé. Revista Portuguesa de Estomatologia, Medicina Dentária e Cirurgia Maxilofacial, 2013; 54: 20-26. |
| Caries prevalence and dental health of 8-12 year-old children in Damascus city in Syria during the Syrian Crisis; a cross-sectional epidemiological oral health survey | 2019 | Syrian Arab Republic/ EMRO | Ballouk MA, Dashash M. Caries prevalence and dental health of 8-12 year-old children in Damascus city in Syria during the Syrian Crisis; a cross-sectional epidemiological oral health survey. BMC Oral Health. 2019 Jan 15;19(1):16. doi: 10.1186/s12903-019-0713-9. PMID: 30646889; PMCID: PMC6332908. |
| The dental health of 5 year-old children living in Damascus, Syria | 2012 | Syrian Arab Republic/ EMRO | Dashash M, Blinkhorn A. The dental health of 5 year-old children living in Damascus, Syria. Community Dent Health. 2012 Sep;29(3):209-13. PMID: 23038936. |
| Using associations between oral diseases and oral health-related quality of life in a nationally representative sample to propose oral health goals for 12-year-old children in Thailand | 2012 | Thailand / SEARO | Krisdapong, Sudaduang & Prasertsom, Piyada & Rattanarangsima, Khanit & Adulyanon, Supreda & Sheiham, Aubrey. (2012). Using associations between oral diseases and oral health-related quality of life in a nationally representative sample to propose oral health goals for 12-year-old children in Thailand. International dental journal. 62. 320-30. 10.1111/j.1875-595x.2012.00130.x. |
| The Association of Socioeconomic Status and Dental Caries Experience in Children in Dili, Timor-Leste | 2015 | East Timor / AFRO | Babo Soares LF, Allen P, Bettiol S, Crocombe L. The Association of Socioeconomic Status and Dental Caries Experience in Children in Dili, Timor-Leste. Asia Pac J Public Health. 2016 Oct;28(7):620-628. doi: 10.1177/1010539516667783. Epub 2016 Sep 24. PMID: 27620835. |
| Goals for oral health in Tunisia 2020 | 2020 | Tunisia / EMRO | Maatouk F, Jmour B, Ghedira H, Baaziz A, Ben Hamouda L, Abid A. Goals for oral health in Tunisia 2020. East Mediterr Health J. 2012 Oct;18(10):1072-7. doi: 10.26719/2012.18.10.1072. PMID: 23301364. |
| National survey of oral health status of children and adults in Turkey | 2009 | Turkey / EURO | Gökalp SG, Doğan BG, Tekçiçek MT, Berberoğlu A, Unlüer S. National survey of oral health status of children and adults in Turkey. Community Dent Health. 2010 Mar;27(1):12-7. PMID: 20426255. |
| The oral health and treatment needs of schoolchildren in Trinidad and Tobago: findings of a national survey | 2006 | Trinidad and Tobago / OPAS | Naidu R, Prevatt I, Simeon D. The oral health and treatment needs of schoolchildren in Trinidad and Tobago: findings of a national survey. Int J Paediatr Dent. 2006 Nov;16(6):412-8. doi: 10.1111/j.1365-263X.2006.00755.x. PMID: 17014539. |
| Ministry of Health and Welfare | 2012 | Taiwan, Province of China / WPRO | https://www.mohw.gov.tw/mp-2.html |
| The Oral Health Situation of 12-Year-Old School Children in the Rural Region of Ilembula in Southwestern Tanzania: A Cross-Sectional Study | 2020 | Tanzania / AFRO | Zumpe L, Bensel T, Wienke A, Mtaya-Mlangwa M, Hey J. The Oral Health Situation of 12-Year-Old School Children in the Rural Region of Ilembula in Southwestern Tanzania: A Cross-Sectional Study. Int J Environ Res Public Health. 2021 Nov 22;18(22):12237. doi: 10.3390/ijerph182212237. PMID: 34831993; PMCID: PMC8618583. |
| Dental Caries Pattern Amongst Tanzanian Children: National Oral Health Survey | 2022 | Tanzania / AFRO | Mbawalla HS, Nyamuryekung'e KK, Mtaya-Mlangwa M, Masalu JR. Dental Caries Pattern Amongst Tanzanian Children: National Oral Health Survey. Int Dent J. 2023 Oct;73(5):731-737. doi: 10.1016/j.identj.2023.03.008. Epub 2023 Jun 10. PMID: 37308399; PMCID: PMC10509444. |
| Oral health status and treatment needs in different age groups in two regions of Tanzania | 2002 | Tanzania / AFRO | Mosha HJ, Ngilisho LA, Nkwera H, Scheutz F, Poulsen S. Oral health status and treatment needs in different age groups in two regions of Tanzania. Community Dent Oral Epidemiol. 1994 Oct;22(5 Pt 1):307-10. doi: 10.1111/j.1600-0528.1994.tb02057.x. PMID: 7813182. |
| Dental pain, oral impacts and perceived need for dental treatment in Tanzanian school students: a cross-sectional study | 2009 | Tanzania / AFRO | Mashoto KO, Astrøm AN, David J, Masalu JR. Dental pain, oral impacts and perceived need for dental treatment in Tanzanian school students: a cross-sectional study. Health Qual Life Outcomes. 2009 Jul 30;7:73. doi: 10.1186/1477-7525-7-73. PMID: 19643004; PMCID: PMC2726126. |
| Systems for the provision of oral health care in the black sea countries. Part 12: Ukraine | 2012 | Ukraine EURO | Marino B, Domenico D, Igmazia C, Denga A, Paganelli C, Dianiskova S. Systems for the provision of oral health care in the black sea countries. Part 12: Ukraine. Oral Health Dent Manag. 2012 Dec;11(4):149-51. Erratum in: Oral Health Dent Manag. 2013 Jun;12(2):119. PMID: 23208591. |
| Prevalence, Severity and Factors Associated with Dental Caries Among School Adolescents in Uganda: A Cross-Sectional Study | 2020 | Uganda / AFRO | Ndagire B, Kutesa A, Ssenyonga R, Kiiza HM, Nakanjako D, Rwenyonyi CM. Prevalence, Severity and Factors Associated with Dental Caries Among School Adolescents in Uganda: A Cross-Sectional Study. Braz Dent J. 2020 Mar-Apr;31(2):171-178. doi: 10.1590/0103-6440202002841. PMID: 32556017; PMCID: PMC8346632. |
| Prevalence and factors associated with dental caries among children and adults in selected districts in Uganda | 2015 | Uganda / AFRO | Kutesa A, Kasangaki A, Nkamba M, Muwazi L, Okullo I, Rwenyonyi CM. Prevalence and factors associated with dental caries among children and adults in selected districts in Uganda. Afr Health Sci. 2015 Dec;15(4):1302-7. doi: 10.4314/ahs.v15i4.33. PMID: 26958035; PMCID: PMC4765403. |
| Oral health surveillance report : trends in dental caries and sealants, tooth retention, and edentulism, United States : 1999–2004 to 2011–2016 | 2016 | United States of America / OPAS | Centers for Disease Control and Prevention. Oral Health Surveillance Report: Trends in Dental Caries and Sealants, Tooth Retention, and Edentulism, United States, 1999–2004 to 2011–2016. Atlanta, GA: Centers for Disease Control and Prevention, US Dept of Health and Human Services; 2019. |
| Dental caries in Uruguayan adults and elders: findings from the first Uruguayan National Oral Health Survey | 2015 | Uruguay / OPAS | Álvarez L, Liberman J, Abreu S, Mangarelli C, Correa MB, Demarco FF, Lorenzo S, Nascimento GG. Dental caries in Uruguayan adults and elders: findings from the first Uruguayan National Oral Health Survey. Cad Saude Publica. 2015 Aug;31(8):1663-72. doi: 10.1590/0102-311X00132214. PMID: 26375645. |
| Perfil epidemiológico bucal de las etnias venezolanas. Primer reporte nacional | 2008 | Venezuela / OPAS | Borjas, Alexis. (2008). Perfil epidemiológico bucal de las etnias venezolanas. Primer reporte nacional. Ciencia Odontológica. 5. 11-. |
| National Oral Health Survey, Vanuatu 2017 | 2017 | Vanuatu / WPRO | https://msm.org.au/gudfala-tut/vanuatu-national-oral-health-survey-2017-nohs-2017/ |
| Oral health status of 12-year-old school children in Yemen | 2012 | Yemen / EMRO | Al-Otaibi MF, Al-Mamari F, Baskaradoss JK. Oral health status of 12-year-old school children in Yemen. A cross- sectional survey. Eur J Paediatr Dent. 2012 Dec;13(4):324-8. PMID: 23270293. |
| Dental Caries, Body Mass Index, and Diet among Learners at Selected Primary Schools in Pretoria, Gauteng Province, South Africa | 2019 | South Africa / AFRO | Nkambule NR, Madiba TK, Bhayat A. Dental Caries, Body Mass Index, and Diet among Learners at Selected Primary Schools in Pretoria, Gauteng Province, South Africa. J Contemp Dent Pract. 2019 Nov 1;20(11):1241-1248. PMID: 31892673. |
| Dental status of children receiving school oral health services in Tshwane | 2019 | South Africa / AFRO | Molete MM, Igumbor J, Stewart A, Yengopal V. Dental  status of children receiving school oral health services  in Tshwane. S. Afr. dent. j. [Internet]. 2019 May [cited  2024 Feb 29] ; 74( 4 ): 171-177.  Available from: [http://www.scielo.org.za/scielo.php ?script](http://www.scielo.org.za/scielo.php%20?script)=sci_arttext&pid=S001185162019000400004&lng=en. <http://dx.doi.org/10.17159/2519-0105/2019>  /v74no4a2. |
| Dental caries in six, 12 and 15 year old Venda children in South Africa | 2004 | South Africa / AFRO | Bajomo AS, Rudolph MJ, Ogunbodede EO. Dental caries in six, 12 and 15 year old Venda children in South Africa. East Afr Med J. 2004 May;81(5):236-43. doi: 10.4314/eamj.v81i5.9166. PMID: 15508337. |
| Dental caries and oral health practice among 12 year old school children from low socio-economic status background in Zimbabwe | 2013 | Zimbabwe / AFRO | Mafuvadze BT, Mahachi L, Mafuvadze B. Dental caries and oral health practice among 12 year old school children from low socio-economic status background in Zimbabwe. Pan Afr Med J. 2013 Apr 29;14:164. doi: 10.11604/pamj.2013.14.164.2399. PMID: 23819006; PMCID: PMC3696470. |
