# Supplementary material for: Quality of evidence in the oral health international data: Contributions for a global profile
Source: PLoS One. 2025 Feb 12;20(2):e0315131. doi: 10.1371/journal.pone.0315131 (PMC11819551; doi:10.1371/journal.pone.0315131)
Supplement: Attachment — Submitted filename: LATTER_ED_PLOSONE2.pdf [file pone.0315131.s002.pdf]

Letter to the Editor

We appreciate all the feedback provided. We acknowledge that the reviewers raised extremely relevant points, and we will address each one individually. We are committed to refining the material to contribute effectively and accurately to the literature on the validation of oral health information.

Reviewer 1:

Comment 1: Page 1, lines 28-29: The sentence appears to end abruptly. Please review.

Response: The authors thank the reviewer for this correction. We agree and have made the necessary change to improve comprehension. (Modified in the text: Page 1, lines 28-29)

Comment 2: Page 4, lines 96 to the end of the page: Could you please add some clarification on why AXIS was specifically chosen, if other frameworks were considered, and what are the strengths and limitations of the framework? Some of these details could be included later in your discussion, but it is good to have this information available.

Response: The authors found it appropriate to include the justification for using the tool in the Methods section. (Modified in the text: Page 4, lines 100-102). We did not find it necessary to include a discussion of other evaluation tools as the AXIS tool was notably distinguished for being current and broadly applicable.

Comment 3: Page 7: There is a remaining comment in your PDF submission. Please check your manuscript carefully for any tracked changes or other issues before resubmitting.

Response: The authors apologize for the inconvenience. The empty comment has been removed, and the corresponding location now has a notation for verification. (Modified in Table 2, after line 165, page 4)

Comment 4: a. Could you please provide some explanation for the variations observed between countries/regions? Currently, your discussion merely repeats the main findings without any critical reflection on why some regions may perform better or worse. This is a missed opportunity. b. There are no clear strengths/limitations (especially the latter) concerning this work. Could you reflect (critically) on your work and share its strengths/limitations?

Response: a. The authors consider this feedback extremely pertinent. We discussed factors that might locally affect the variation in continental and regional scores but lacked a conclusion on the determinants of data production conditions. Consequently, a paragraph has been added to address this. (Modified in the text, lines 250-267, Pages 10-11) b. The authors acknowledge that the final paragraphs were reduced in clarity during translation. Key points required revision due to their importance for concluding the argument and impact on the science, so they have been rewritten. (Modified in the text, lines 317-322, 323-331, Pages 12-13)

Reviewer 2:

Comment 1: The rationale for conducting this analysis needs to be highlighted in the introduction.

Response: The authors recognize a translation error that diminished the previously elaborated rationale and sincerely apologize. The paragraph has been rewritten to clearly state the justification for the study based on the literature gap and social impact. (Modified in the text, lines 65-71, Page 3)

Comment 2: The study relies exclusively on secondary data from the CAPP database, which inherently limits control over data quality. The authors acknowledge this but do not provide

sufficient insights into how data quality issues may distort specific findings or how to mitigate this in future studies.

Response: The authors are committed to studying the database in detail. The quality of evidence is part of a series of articles being developed, with this study serving as the starting point. Data quality indeed distorts oral health records, and a subsequent article will focus exclusively on analyzing documents considered well-rated by the AXIS tool, offering a comparative overview between the results obtained and previous studies. We hope to clarify why this topic was not addressed in the current study. Additionally, the need to counteract the production of low-quality data is emphasized not only through investment in oral health research but also through adopting selection criteria based on methodology and study representativeness. Sometimes a well-developed study does not represent the target population, affecting the generalization of results. (Modified in the text, lines 310-316, Page 12)

Comment 3: Geographic representation: A critical weakness of the article is the uneven geographic representation of data quality. Although this is a limitation of the CAPP database, the study could have provided more solutions to address this issue. The lack of continuous and updated data in certain regions, particularly in Africa and Southeast Asia, further weakens the generalization of findings.

Response: The authors agree with this observation and highlight the geographical concentration as a limiting factor in the final paragraphs. We attempted to include as much data as possible, analyzing studies from 24 years ago to the most recent ones. While temporal comparison is not observed in all scenarios, what has been reported is significant. Some solutions were considered for the absence of data, but to ensure reliability, we worked only with the original data collected. Regional grouping was one attempt to observe if regions evolved differently over the years. Future studies have partially addressed this by weighting the article scores based on population numbers, aiming to clarify territorial representativeness, though it was not generalized across all territories within a region.

Comment 4: a. Data Quality by Region: The results indicate significant variation in data quality across regions, with the Americas and Western Pacific regions having better scores compared to Africa and Southeast Asia. This analysis is useful, but the article is insufficient in discussing the potential sociopolitical factors behind these disparities. A more in-depth exploration of why certain regions perform better or worse could add value to the analysis. b. Impact of Age Groups and Time Periods: The study concludes that records focusing on children and young adults and more recent studies have higher quality data. However, the reasons for this are not fully explored. The authors suggest that this may be due to WHO guidelines focusing on younger age groups but do not delve into a deeper analysis.

Response: a. The authors clarify that the in-depth exploration of regional performance has been developed in subsequent works, where political, socioeconomic implications, and relationships with the development of the health system were explored. We apologize if this gap caused any difficulties in connecting the discussion, but the intention was to introduce a more robust discussion through this mapping. b. We will base our response on subsequent studies that examine oral health data from CAPP using only reliable data and compared to the literature. We have detailed the importance and implications of studies focusing on children around the age of 12. We apologize if this gap affected the study and affirm that we look forward to feedback on these responses and are fully open to considering the editor's opinions. We appreciate the valuable contributions.

Comment 5: Study Limitations: The authors acknowledge the main limitations, such as the uneven quality of CAPP data and the use of secondary sources. However, there are additional limitations that could have been addressed more thoroughly:

- a. Lack of in-depth analysis of regional disparities: The article identifies disparities in data quality but does not propose robust solutions to address these gaps. For example, the study could have suggested methodologies to improve data collection in underrepresented regions.
- b. No validation of data from original sources: The study does not attempt to cross-validate the data included in the CAPP database with other data sources, which could have strengthened the findings. Relying solely on the CAPP database limits the study's ability to assess the true reliability of the data.

Response: a. The authors note that methodological shortcomings are a result of underinvestment due to a lack of financial resources or prioritization, which is why new methodologies beyond detailing the most significant methodological issues were not explored. b. The authors focused exclusively on CAPP due to its significant importance. However, with the publication of evaluations from this database, we hope to develop comparative studies with other sources. The research was extremely detailed and involved evaluating various scenarios. Ideally, similar evaluations should be conducted for other data catalogs to guide researchers in choosing the database that best aligns with their study objectives and to address data quality issues, as in the case of applying the AXIS tool.

Comment 6: Implications and Practical Recommendations: Although the study provides a valuable critique of the CAPP database, it lacks actionable recommendations for policymakers. Suggestions such as implementing more rigorous inclusion criteria, improving data collection in underrepresented regions, or fostering international collaboration to enhance the database would have been beneficial.

Response: The authors attempt to address this critical need throughout the manuscript by noting that regions with significant investments have better health data. The final pages (Modified in the text, Pages 12 and 13) now include the need for stricter inclusion criteria to avoid generalizing results that impair policy development and scientific evidence, and emphasize the importance of prioritizing oral health research to foster international collaborations for better and new data.

Sincerely,

The Authors
